# Supplementary material for: The development, feasibility and credibility of intra-abdominal pressure measurement techniques: A scoping review
Source: PLoS One. 2024 Mar 21;19(3):e0297982. doi: 10.1371/journal.pone.0297982 (PMC10956852; doi:10.1371/journal.pone.0297982)
Supplement: S1 File — (DOCX) [file pone.0297982.s001.docx]

**S1 File. Search strategies of each database**

| Database | Literature | Search Strategy |
| --- | --- | --- |
| Pubmed | 2563 | (("[intra-abdominal pressure](https://www.ncbi.nlm.nih.gov/mesh/68060305)"[MeSH Terms] OR ((((("intraabdominal pressure" [Title/Abstract] OR "bladder pressure"[Title/Abstract]) OR "intravesical pressure"[Title/Abstract]) OR "intra-vesical pressure"[Title/Abstract]) OR "intra-vaginal pressure"[Title/Abstract]) OR "vesicular pressure"[Title/Abstract]) OR "intra-gastric pressure"[Title/Abstract]) OR "femoral venous pressure" [Title/Abstract]) OR "rectal pressure" [Title/Abstract])))) AND (("methods"[MeSH Terms] OR "measure*" (((([Title/Abstract]) OR "measuring"[Title/Abstract]) OR "measurement*"[Title/Abstract]) OR "monitor*"[Title/Abstract]) OR "technique*"[Title/Abstract])) OR "technology"[Title/Abstract])))) Filters: From 2000-2023, Full text, Humans, English. |
| Embase | 1625 | (('[intra-abdominal pressure](https://www.ncbi.nlm.nih.gov/mesh/68060305)'/exp) OR( 'intraabdominal pressure' OR 'bladder pressure' OR 'intravesical pressure' OR 'intra-vesical pressure' OR 'intra-vaginal pressure' OR 'vesicular pressure' OR 'intra-gastric pressure' OR 'femoral venous pressure' OR 'rectal pressure':ti)) AND ('method*'/exp OR 'measure*'/exp OR 'measuring'/exp OR 'measurement*'/exp OR 'monitor*'/exp) OR 'technique*'/exp OR 'technology'/exp) |
| Ebsco | 1804 | TI([intra-abdominal pressure](https://www.ncbi.nlm.nih.gov/mesh/68060305) OR intraabdominal pressure OR bladder pressure OR intravesical pressure OR intra-vesical pressure OR intra-vaginal pressure OR vesicular pressure OR intra-gastric pressure OR femoral venous pressure OR rectal pressure) AND AB (method* OR measure* OR measuring OR measurement* OR monitor* OR technique* OR technology) |
| Proquest | 247 | ti([intra-abdominal pressure](https://www.ncbi.nlm.nih.gov/mesh/68060305) OR intraabdominal pressure OR bladder pressure OR intravesical pressure OR intra-vesical pressure OR intra-vaginal pressure OR vesicular pressure OR intra-gastric pressure OR femoral venous pressure OR rectal pressure) AND ab(method* OR measure* OR measuring OR measurement* OR monitor* OR technique* OR technology) filter:from 2000-2023, full-text |
| Scopus | 2373 | ( TITLE-ABS-KEY (intra-abdominal AND pressure OR intraabdominal AND pressure OR bladder AND pressure OR intravesical AND pressure OR intra-vesical AND pressure OR intra-vaginal AND pressure OR vesicular AND pressure OR intra-gastric AND pressure OR femoral AND venous AND pressure OR rectal AND pressure) AND TITLE-ABS-KEY ( method* OR measure* OR measuring OR measurement* OR monitor* OR technique* OR technology) |
| Web of Science | 1336 | TI([intra-abdominal pressure](https://www.ncbi.nlm.nih.gov/mesh/68060305) OR intraabdominal pressure OR bladder pressure OR intravesical pressure OR intra-vesical pressure OR intra-vaginal pressure OR vesicular pressure OR intra-gastric pressure OR femoral venous pressure OR rectal pressure) AND TI (method* OR measure* OR measuring OR measurement* OR monitor* OR technique* OR technology) |
