# Supplementary material for: The development, feasibility and credibility of intra-abdominal pressure measurement techniques: A scoping review
Source: PLoS One. 2024 Mar 21;19(3):e0297982. doi: 10.1371/journal.pone.0297982 (PMC10956852; doi:10.1371/journal.pone.0297982)
Supplement: S2 File — (DOCX) [file pone.0297982.s002.docx]

**S2 File. Summary of measurement methods**

1. **Staelens et al. (2023)**
2. Equipment

A rectal T-DOC 7Fr airflled balloon catheter (Laborie Medical Technologies, Mississauga, Canada) connected to a computer displaying the IAP (Audact Pro database version 7.11, Ellipse Andromeda, Urotex, The Netherlands) (ESM Fig. 1).


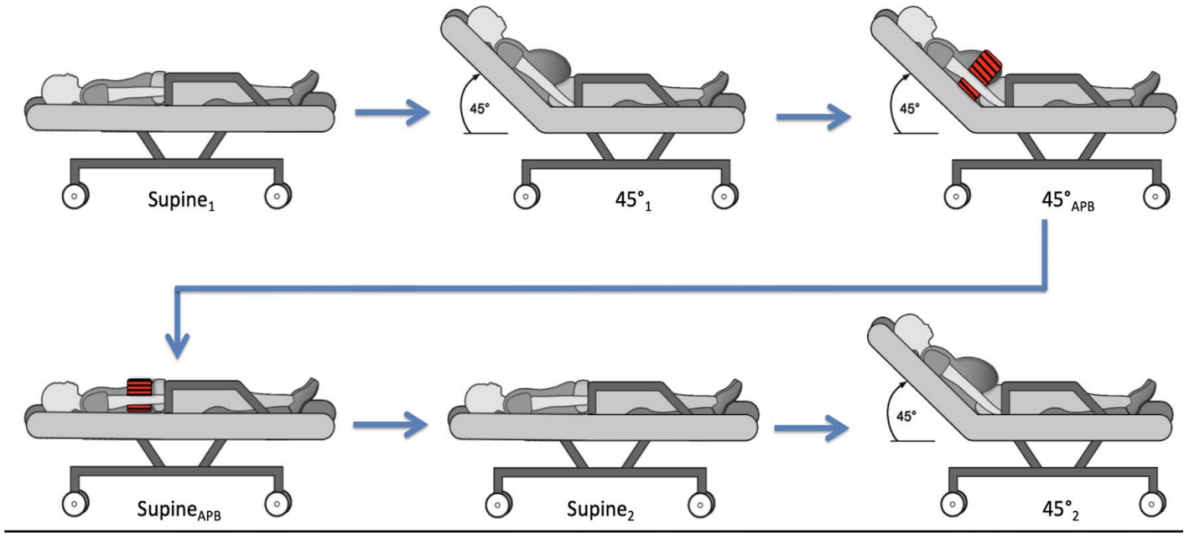


**Fig 1.** Standardized protocol to measure intra-abdominal pressure. Intra-abdominal pressure measurements were performed in diferent positions: supine position (Supine1 and Supine2) and 45° semirecumbent (45°1 and 45°2) without an external abdominal pressure belt, and 45°semirecumbent (45°APB) and supine position (SupineAPB) with an external abdominal pressure belt (marked with a red spot)

（2）Measurements

The balloon was infated with air using a switch, zeroed at atmospheric pressure and inserted 15 cm into the rectum after digital rectal palpation to remove impacted faeces. The catheter was attached to the patient’s leg to prevent displacement.To validate the accuracy of IAPrect with increasing IAP, measurements were performed in 2 positions in an attempt to artifcially increase IAP; the 45°elevated head of bed semirecumbent position, followed with an external abdominal pressure belt (similar to that used by surgeons to prevent incisional hernias). The abdominal belt was put on manually and fastened with a velcro tape and was not released during the protocol. IAPves and IAPrect were measured simultaneously according to a standardized protocol (Fig. 1). All positions (except the application of the external abdominal pressure belt) were repeated twice, including the insertion of the rectal catheter.

1. **Abassi et al. (2018)**
2. Equipment

DantecMenuet equipment (M 247) and fluid-filled lines with external pressure transducers.

（2）Measurements

An 8 F double-lumen pressure catheter was introduced into the urinary bladder transurethral under sterile conditions. One lumen was used to evacuate the bladder and infuse 50 ml of normal saline at room temperature. The other lumen was used for pressure measurement. A 10 F single-lumen pressure catheter with a balloon tip connected to a pressure transducer was then introduced through the epigastric port and the balloon tip is placed intraperitoneally under direct vision. The catheter has a 3-way extension through which the balloon is filled with normal saline and after evacuation of air the fluid is retained in the syringe. Once the catheter is placed in the abdomen the fluid is flushed back into the balloon. The transducer tubings are infused with normal saline to evacuate the air and calibrated to zero pressure before connecting to the urethral pressure catheter. All pressure measurements were performed with the patients lying in supine position under general anesthesia before starting laparoscopic cholecystectomy.

The level of the symphysis pubis has been chosen for calibrating the pressure transducers for its simplicity to localize. Zero balancing (calibrating the transducer’s output signal at zero pressure) was performed at atmospheric pressure with a stabilizing period of about 3 min before taking pressure measurements. Once the connections are made, to avoid any artifacts, the pressure lumen catheters are flushed with a minimal amount of saline through the transducer channel to avoid any air bubble or gel interruption. The baseline IAP and IBP measurements are pressures recorded when no air insufflations applied to the abdomen. The two pressure measurements were then simultaneously recorded by stepwise increment of IAP by increasing the insufflation pressure gradually to approximately 22 mmHg, as shown on the laparoscopy CO2 insufflator. The readings are detected by Dantec Menuet equipment and documented internally as the pressure goes up.

1. **Kusar et al. (2022)**

（1）Equipment

Using transcutaneous sensors (MC-System, TMG-BMC, Ljubljana, Slovenia) to measure the

tension on the abdominal wall. Te sensor’s indenting tip protrudes towards the skin surface, where the force on the tip changes with the tension on the surface. Te piezoresistive silicon force sensor with known sensitivity returns the output signal proportional to the indenting force. Te electric activity of the abdominal muscles was measured with a surface bipolar EMG electrode (Skintact

F-301, Leonhard Lang GmbH, Austria).

1. Measurements

The volunteers were placed in a supine position with the bed levelled without inclination.

Four sensors were placed (one on the cranial end of each rectus muscle and one on each oblique muscle, in the area between the anterior and middle axillary lines) and further secured with kinesiology tape. Two EMG sensors were placed on the caudal end of one of the rectus muscles, with the ground electrode placed near the iliaccrest. Te ideal positioning of the electrodes is shown in Fig. 1. The precise positions of the sensors (as well as the side of the EMG electrodes) were adjusted to accommodate the patients’ operative incisions, drains and ostomies (in patients with ostomies/drains/nonmedian incisions, the EMG electrodes were placed contralaterally, while

the ipsilateral MC electrode positions were adjusted as needed, always overlying the corresponding muscle). Afer attachment of the sensors, a 2 min period was used to neutralise the efect of skin viscoelasticity. The indentation depth of the transcutaneous sensor tip was set to 6 mm.

The IAP baseline, transcutaneous sensor signals and EMG signals were measured during calm breathing with relaxed abdominal muscles. The volunteers were then asked to perform the Valsalva manoeuvre 4 times, with at least 30 seconds between attempts to ensure a fall of IAP towards baseline. Patients were asked to maintain each attempt until the meniscus of urine/saline solution in the measurement system equilibrated, allowing for a reading of the intravesical IAP value on the scale. Peak intravesical IAP values under tension and resting intravesical IAP values were recorded for each attempt, resulting in 4 values under tension and 4 values during rest for each patient. Afer removal of the measurement systems, ultrasound of the abdominal wall was performed. We recorded the maximum rectus thickness and overlying subcutaneous tissue thickness in the area of the upper rectus where sensor 1 had previously been placed. Whole abdominal wall thickness was calculated as the sum of the two.


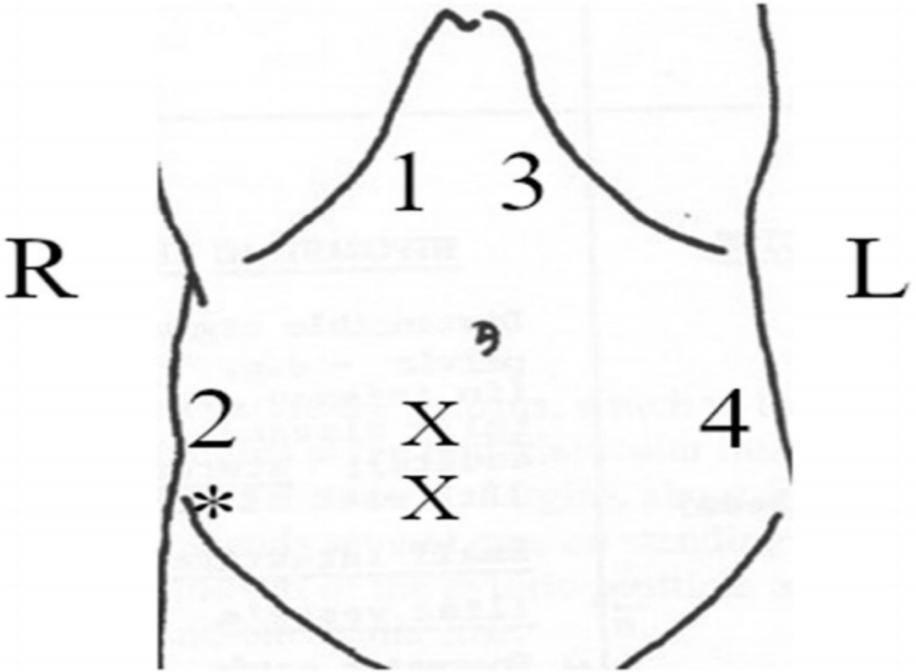


**Fig 2.** Idealised placement of MC and EMG sensors on the abdomen. Numbers 1–4 represent the placement of MC sensors; US measurements were conducted at 1. Te double X shows the placement of both EMG electrodes. * shows the placement of the EMG ground electrode.

1. **Tang et al. (2021)**
2. Equipment

A noninvasive multipoint abdominal wall tension measurement device (Chinese patent No. ZL 201510799207.4) was used. The device consisted of a high-precision resistance strain pressure transducer (JHBM-H3 pressure transducer from Bengbu Transducer System Engineering Co., Ltd, China), a displacement sensor with self-reset spring (KTR-A self-reset linear displacement sensor from Taizhou Quantum Electronic Technology Co., Ltd, China), and a data informationprocessing module (STMicroelectronicsSTM32 microcontroller, Italy; 24-bit AD conversion chip HX711, Avia Semiconductor Co., Ltd, China) (see Fig 3)

1. Measurements

Designating a dedicated person to perform AWT measurement and data collection. Using human bone markers (xiphoid process, symphysis pubis) and fixed structures (navel) as the standardization points for AWT measurement points and follow the standard AWT measurement point determination method by using a marker to mark the surface of the patient’s abdominal wall, as shown in Fig 4.

Patients were kept in a quiet state, in a supine position, with clothing, accessories and other coverings on the abdomen removed. For patients who were mechanically ventilated, the ventilator parameter positive end expiratory pressure (PEEP) was adjusted to 0 mmHg before each measurement. After turning on the switch of the device, the pressure gauge was placed vertically on the surface of the measurement point of the abdominal wall, and the displacement sensor was pressed to the maximum displacement distance (5 cm) at a constant speed at the end of expiration. Each measurement lasted for 2–3 s, and the same method was used to measure each point approximately 20–30 times. Then, the device switch was turned off, and the measurement was completed.


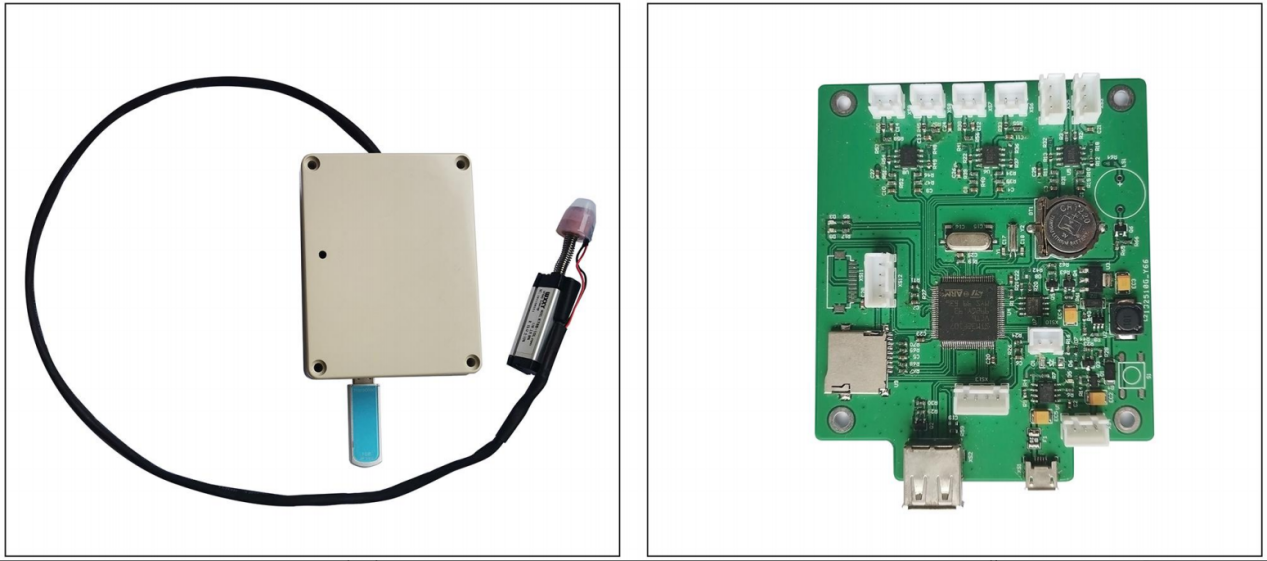


**Fig 3.** Appearance of the AWT measuring device


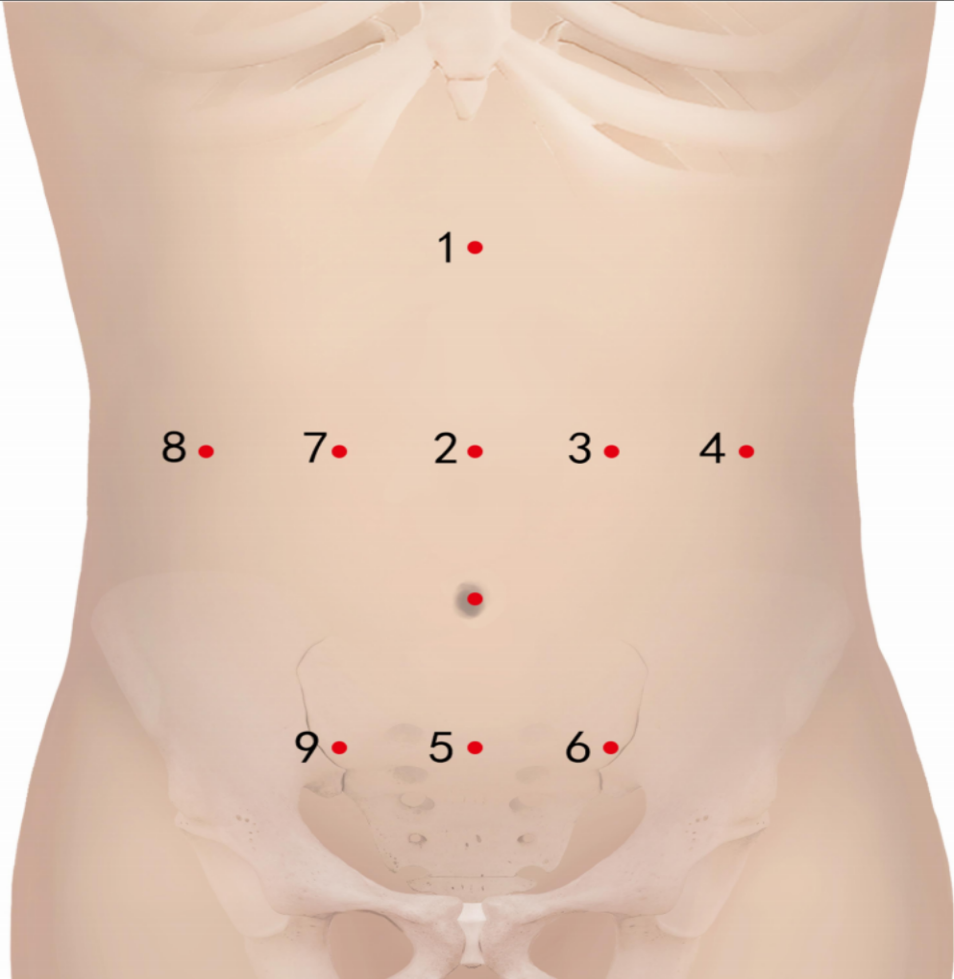


**Fig 4.** Body surface location of the 9 points on the abdominal wall. Point 1: 5 cm below the xiphoid process; Point 6: 5 cm to the left of Point 5. Point 2: 5 cm above the belly button; Point 7: 5 cm to the right of Point 2. Point 3: 5 cm to the left of Point 2; Point 8: 10 cm to the right of Point 2. Point 4: 10 cm to the left of Point 2; Point 9: 5 cm to the right of Point 5. Point 5: 5 cm above the symphysis pubis.

1. **Jacobson et al. (2022)**
2. Equipment

A novel device as described by Jacobson and Driscoll and shown in Fig 5.The novel device induces a suction (app) against the skin from which the resulting tissue deformation (*w*) is measured by onboard sensors for pressure (BMP388, Adafruit) and distance (VL6180, Adafruit), respectively. The maximum lateral deformation reading of the VL6180 is 100 mm, with a maximum standard deviation of 2 mm across 100 measurements at 50 mm (4%). The operating range of the BMP388 is 225 to 937 mmHg with a relative accuracy of ±0.06 mmHg.


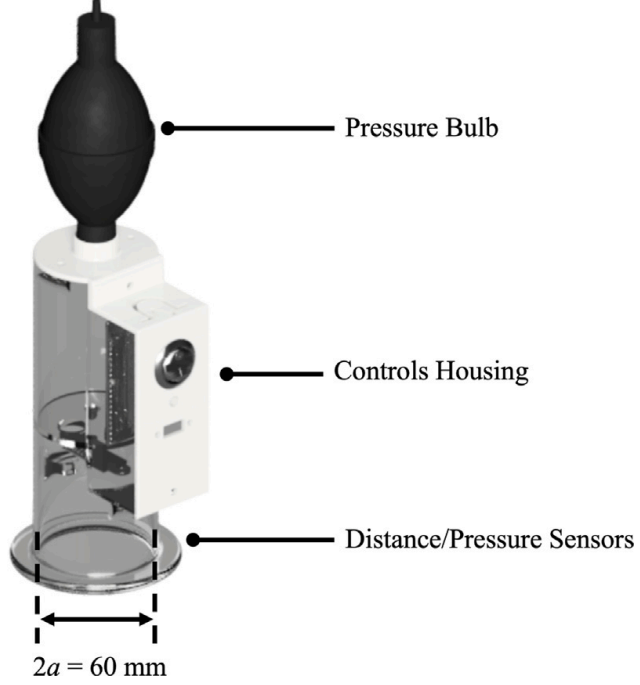


**Fig 5.** Prototype novel device with major components denoted and radius (*ɑ*)identified (Jacobson and Driscoll, 2020)

1. Measurements

Anatomic geometry was measured with US in living participants for AWTh 5 cm subxiphoid, while a soft measuring tape was used to determine abdominal circumference at the navel. The inner and outer radii of participants was calculated assuming the torso to have a circular cross-section. US jelly was used between the device and skin to improve airtightness and resulting suction. Three suction pulses were applied, from which the average IAP was calculated. Due to poor sensitivity of Eq. (2) to changes in body position, a fluid pressure factor (*pgh*) was considered as a supplementary value. IAP was determined using the adjusted equation:

IAP= P_in_ + *pgh*

where in is the pressure calculated using suggestions (see Eq. (2)), and is a modifier dependent on body position. As the human body is largely comprised of water, particularly in the abdomen with rates ranging from 60% (reported in connective tissues) to 96% (at the bladder), the density () of the abdomen was approximated as 997 kg/m3. The force of gravity, , was 9.807 m/s2 , while height (ℎ) calculations were based on anthropometric relations (Drillis and Contini, 1966) yielding:

ℎ = 0.095H*sin*(*a*)

for inclined and sitting positions, where is body height, and is the angle of inclination (in rad). To note, 0.095 refers to the relative distance between the hips and abdominal midpoint: halfway between the hips and chest (Drillis and Contini, 1966). The midpoint was selected to identify the average fluid pressure within the IAV. For standing positions,

ℎ = 0.145H

where 0.145 refers to the relative distance between the pelvic crease and the abdominal midpoint. To illustrate anthropometric relations. IVP outputs a fluid height that is directly translated to IAP in mmHg. In each cadaver, 25 mL was directly injected into the bladder via a urinary catheter, and the resulting pressure was measured by a physical manometer setup.

Part 1: Cadavers

Cadavers were in supine position with an inline catheter available. The novel device was used 5 cm subxiphoid,IVP was measured concurrently.

(1) Two measurements (M1a and M2a, Fig. 6) for IAP were taken with the novel device with 2- to 5-min washout periods (time interval between tests). A second researcher repeated the measurement (M3a) immediately after M2a.

(2) Both the novel device and IVP were tested simultaneously as the devices did not affect one another. IVP readings (IVP1a and IVP2a) were taken at the same time as the novel device measurement was read.

(3) The entire procedure was immediately repeated at a head tilt of 25◦ resulting in 3 additional measurements per test method, denoted as M1b through M3b and IVP1b through IVP2b.

Part 2: Living participants

Living participants were in supine position, with tests conducted at end-expiration and without any abdominal activation. The novel device was used 5 cm subxiphoid.

(1) Two measurements (ML1a and ML2a) for IAP were taken with the novel device with 2- to 5-min washout periods (time interval between tests). A second researcher repeated the measurement (ML3a) immediately after ML2a.

(2) As no control device was used, novel device measurements were compared to published average values for IAP.

(3) The entire procedure was immediately repeated at (b) a head tilt of 25◦ , (c) sitting and (d) standing positions, resulting in 9 additional measurements, denoted as ML1b through ML3d. Fig. 6 illustrates the procedure for Part 1 and Part 2.


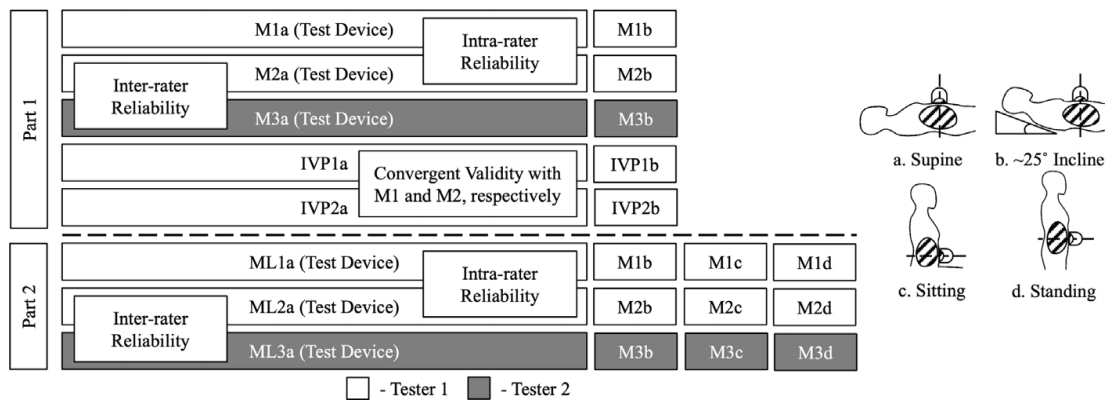


**Fig 6.** Illustrated procedure for Part 1 (cadavers) and Part 2 (living participants) of the present study. Tested anatomical positions are pictured, right: a. Supine, b. Inclined, c.Sitting, d. Standing where c. and d. only applied to living participants. Intra-vesical pressure (IVP) was only measured in cadaveric specimen.

1. **Chen et al. (2015)**
2. Equipment

The prototype AWT measurement instrument includes one thrust meter (Aidebao Instruments Co. Ltd., Zhejiang, China) and one self-made device (Fig 7). This instrument can measure the required thrust to produce displacement (mm). AWT was described as thrust/ displacement (N/mm).

1. Measurements

Choosing the location of 5 cm subxiphoid as the best point for measuring AWT. The measurement was performed once the patients were in a stable and quiet condition in the ICU. The AWT measuring device and AbViserAutoValve apparatus were used to measure the AWT and UBP in supine and 30° supine positions during late inspiration and expiration respectively.


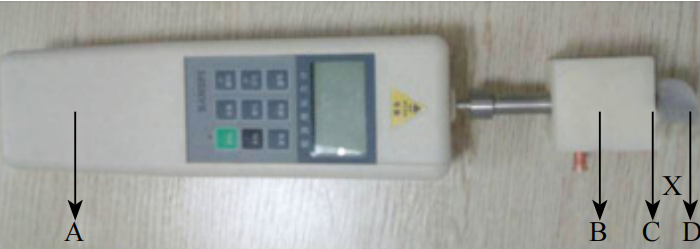


**Fig 7.** The device used for the abdominal wall tension (AWT) measurements. This instrument can measure the required thrust to produce displacement (mm). A: thrust meter; B: self-made device; D: ovalhead. Vertical pressure during the measurement drives the device to the abdominal wall and stops when C is touched by the abdominal wall. X is the vertical displacement of the abdominal wall sag. The thrust is recorded by A. AWT is defi ned as thrust/displacement (N/mm).

1. **Thangarasa et al. (2019)**
2. Equipment

A handheld Stryker pressure monitor connected to the Peritoneal dialysis catheter.

1. Measurements

Standard cannulation of the abdomen by the surgeon was undertaken followed by insufflation, insertion of trocars, and placement of the PD catheter. The superficial cuff and the remainder of the PD catheter were tunneled under the subcutaneous tissue with an exit site in the right or left lower quadrant, flushed, and then connected to a transfer set. The PD transfer set was connected to the sterile fluid path of the STIC pressure monitor system via extension tubing without using the intervening needle. With the patient at end-expiration, the pressure measured from the PD catheter at the level of the umbilicus in the midaxillary line was compared with the insufflator at 15, 10, and 5 mmHg as these pressures were felt to be safe in the context of abdominal surgery.

1. **Howard et al. (2016)**
2. Equipment

An AbViser 300 or 611-kit (Convatec Medical, Greensboro, NC, USA, formerly provided by WolfeTory Medical, Salt Lake City, UT, USA); pressure transducers; a specially engineered transducer device (Fremantle Hospital Medical Equipment Department, Fremantle, WA, see Fig 8); electronic monitoring equipment (Philips IntelliVue MP40, Philips Healthcare, North Ryde, NSW)


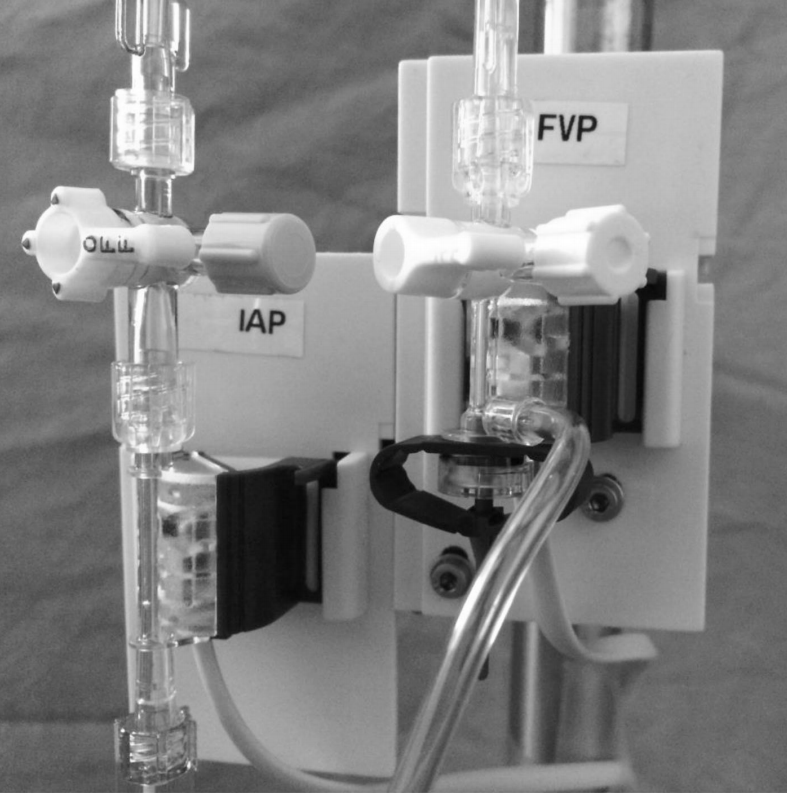


**Fig 8**: Pressure transducer–levelling device.

1. Measurements

Once the patient was enrolled, IAP was measured according to the WSACS consensus recommendations using the standard bladder technique1 . The IAP was measured through the patient’s indwelling catheter, according to the modified Kron technique using an AbViser 300 or 611-kit (Convatec Medical, Greensboro, NC, USA, formerly provided by WolfeTory Medical, Salt Lake City, UT, USA). The transducers for IAP and FVP were zeroed at the level of the superior iliaccrest where the mid-axillary line crosses the iliac crest. The pressure transducers were placed into a specially engineered transducer device (Fremantle Hospital Medical Equipment Department, Fremantle, WA,) and then connected to the electronic monitoring equipment (Philips IntelliVue MP40, Philips Healthcare, North Ryde, NSW).

After zeroing, the pressure was measured at end-expiration in mmHg at the same time as the IAP measurement. For IAP measurement, 20 ml of sterile saline was injected into the bladder through the indwelling catheter; IAP was measured approximately 30 seconds later at end-expiration in mmHg. End-expiratory diastolic and mean venous pressures were recorded using the FVP measurement set to pulmonaryartery pressure (PAP) and using the wedge pressure waveform application on the monitor.

If the baseline IAP measurement was >20 mmHg, no external weight was placed onto the patient’s abdomen and the procedure was repeated for the second set of measurements no less than two hours, and no more than 24 hours later (see Figure 9). If the first IAP was <20 mmHg, further measurements were obtained after placement of an external weight of 5 kg or more (maximum 10 kg) onto the patient’s abdomen. One to two 5 litre bags of dialysate (Accusol 35 solution for haemofiltration, Baxter Healthcare, NSW) were placed in the centre of the abdomen directly over the umbilicus in a horizontal orientation (see Figures 10 and 11).

The IAP and FVP measurements were repeated with 5 kg weight as described above and recorded. If the IAP remained below 20 mmHg with the 5 kg external weight, the measurements were repeated with 10 kg weight as described above, and IAP and FVP were recorded. The same process was followed for the second set of measurements no less than two hours, and no more than 24 hours later.


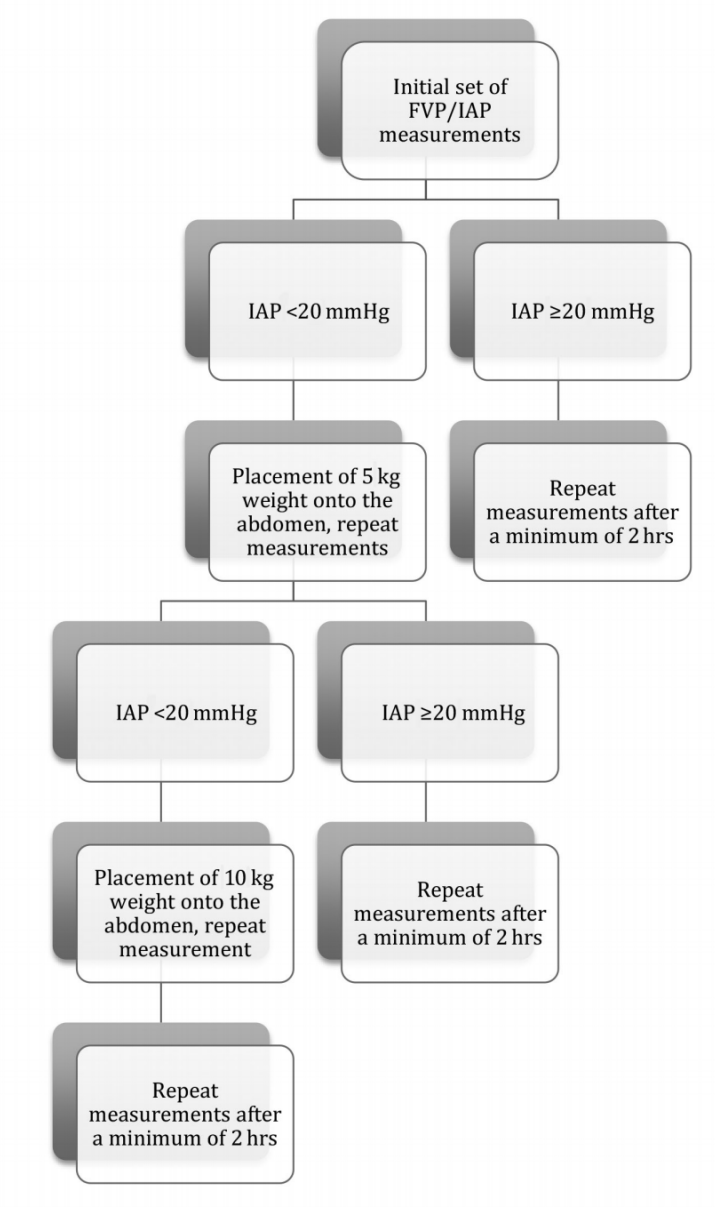


**Fig 9:** Method for the application of external weight.


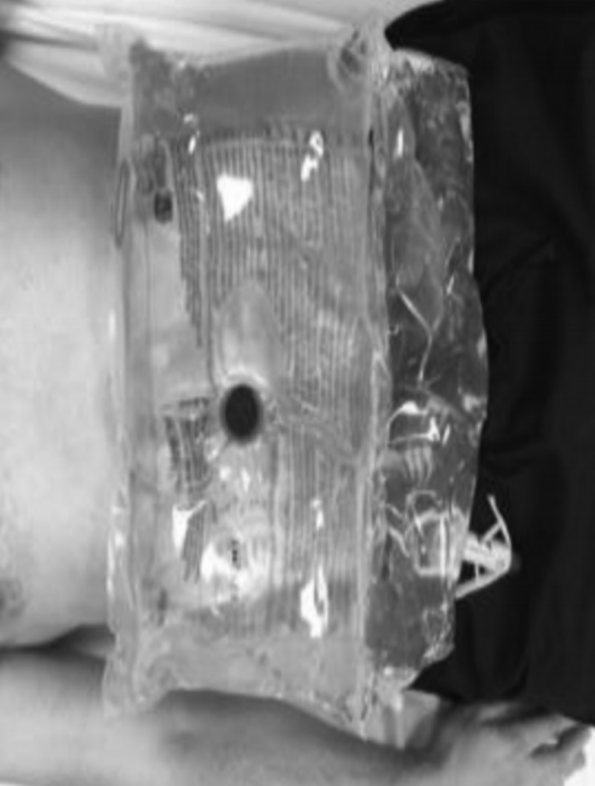

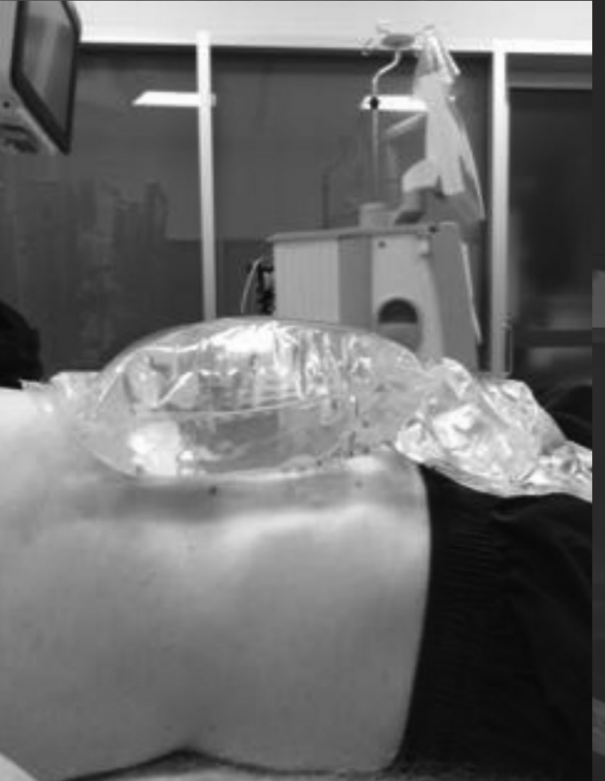


**Fig 10 & 11:** Application of external weight.

1. **Kaussen et al. (2021)**
2. Equipment

Air-capsule-based measurement (ACM-IGP, Spiegelberg company, Germany).

1. Measurements

IGP was determined by air-capsule-based measurement (ACM-IGP, Spiegelberg company, Germany) using a commercially available 9 French double-lumen nasogastric tube catheter with one separate lumen for continuous IGP measurement. A thin air capsule (sized 10×3×2.3 mm) at the tip of the catheter is connected to a pressure transducer of a bedside ACM-IGP monitor. For IAP measurement, the air capsule is flled with a defned air volume of 0.05–0.10 ml through the ACM-IGP catheter lumen. Any variation of external pressure is immediately transduced via the air capsule to the monitor and converted into an electrical signal. Te exact underlying technical process for IAP quantifcation is proprietary and at the discretion of the manufacturer.

The ACM-IGP system has been approved and CE-certifed as an independent medical device for many years; all necessary tests were performed and its biocompatibility and endurance were confrmed ("shelf test"). Te shelf life of the polyurethane catheter is specifed by the manufacturer for 30 days. For anatomical reasons, ACM-IGP catheters can generally be used for patients with a body weight of 3 kg or above. All ACM-IGP catheters were inserted nasally or perorally like conventional nasogastric tubes.

Using sonography, the correct positions of ACM-IGP and transurethral catheters were checked at least daily and additionally whenever ACM-IGP or IVP measurements showed no respiratory undulations. Such respiratory variations are considered as a quality criterion for IAP measurement; their absence indicate a malposition of the ACM-IGP or bladder catheter and usually require their reinsertion.


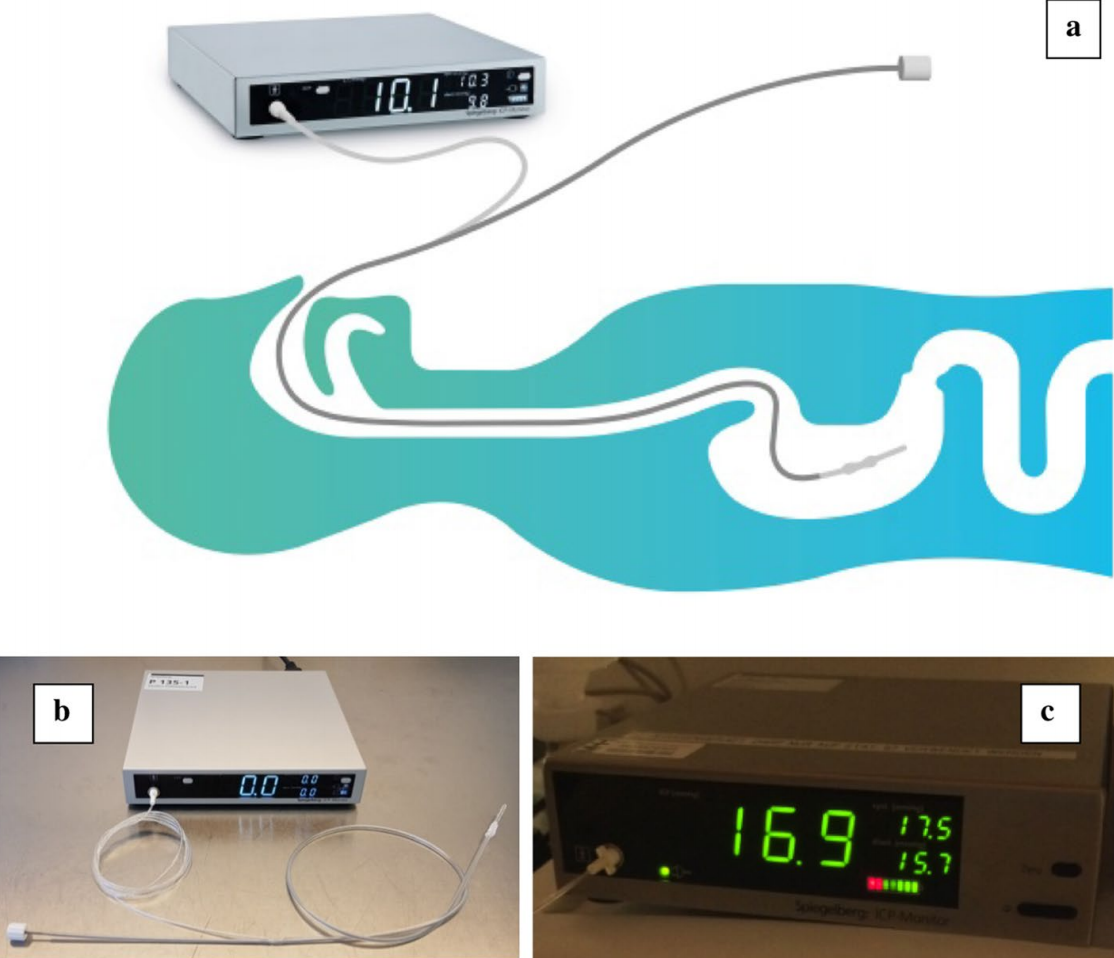


**Fig 11.**Illustration of air-capsule-based intra-abdominal pressure measurement system (ACM-IGP). **a** Schematic illustration of the customized catheter of the air-capsule-based measurement of intra-gastric pressure (ACM-IGP) system, which is equivalent to a special double-lumen 9F nasogastric tube, inserted into the stomach and connected to an ACM-IGP monitor (Illustration courtesy of Spiegelberg Company, Hamburg, Germany). The IAP normally undulates breath-synchronously (here: minimum 9.8 mmHg in expiration, maximum 10.3 mmHg in inspiration and 10.1 mmHg on average). Respiratory variations are considered as a quality criterion for IAP measurement; their absence indicates a malposition of the ACM-IGP or bladder catheter and usually requires their reinsertion. The insertion of an ACM-IGP catheter does not difer from that of a conventional nasogastric tube and is theoretically associated with a similar risk profle (malposition with aspiration, pneumonia, pneumothorax and esophageal or gastric perforation). In patients beyond infancy, the placement is facilitated by an intraluminal guidewire provided by the manufacturer. In neonates and infants, the ACM-IGP catheter was placed without a guidewire, as the narrow bendig of the rigid guidewire in the pharynx hampers a later removal in this age group. All currently available ACM-IGP catheters do not have a radiopaque contrast. Therefore, the gastric catheter location was additionally verifed by abdominal sonography in the present study. **b** Figure illustrates the ACM-IGP atheter connected to the ACM-IGP monitor. On the right side, the white, thin-skinned air capsule (sized 10×3×2.3 mm) is displayed at the gastric end of the ACM-IGP catheter, which is used for IAP measurement. The opposite side is connected to the pressure transducer on the left front of the ACM-IGP monitor. In the left lower margin the guide wire for insertion of the ACM-IGP catheter is displayed on the aboral end of the second lumen. Calibration and "zeroing" of the ACM-IGP system are fully automatic and repeated once per hour in the operating mode. During the continuous IAP measurement, the air capsule is flled with a defned air volume of 0.05–0.10 ml. Any pressure applied to the air capsule from outside is registered by the pressure transducer in the monitor and displayed as IAP with a precision of one decimal. c Illustration of a representative ACM-IGP measurement in a critically ill child with intra-abdominal hypertension (IAH) grade III (IAP=16.9 mmHg). Please note that the displayed pressures with the minimum in exspiration (15.7 mmHg) and maximum in inspiration (17.5 mmHg) represent respiratory variations of IAP

**10. Van et al. (2014)**

1. Equipment

①GastroPV (Holtech Medical, Charlottenlund, Denmark): A new device, the GastroPV is inserted in between the nasogastric probe and the enteral nutrition feeding pump and tubing. The IAP can be estimated via the stomach (i.e. IGP) with the new device (Fig. 12).

②Classic GRV measurement (GRVclassic). The measurement of GRV is neither standardized nor validated. Gastric volume can be considered high if a single volume exceeds 200 mL. The gold-standard up to now, is measuring the GRV by aspiration via a 60 mL syringe after disconnection of the nasogastric tube (GRVclassic) (Fig. 13).

③New GRV measurement (GRVprototype). We used in this study also the GastroPV device to measure the GRV (GRVprototype)(Fig. 14).

1. Measurements


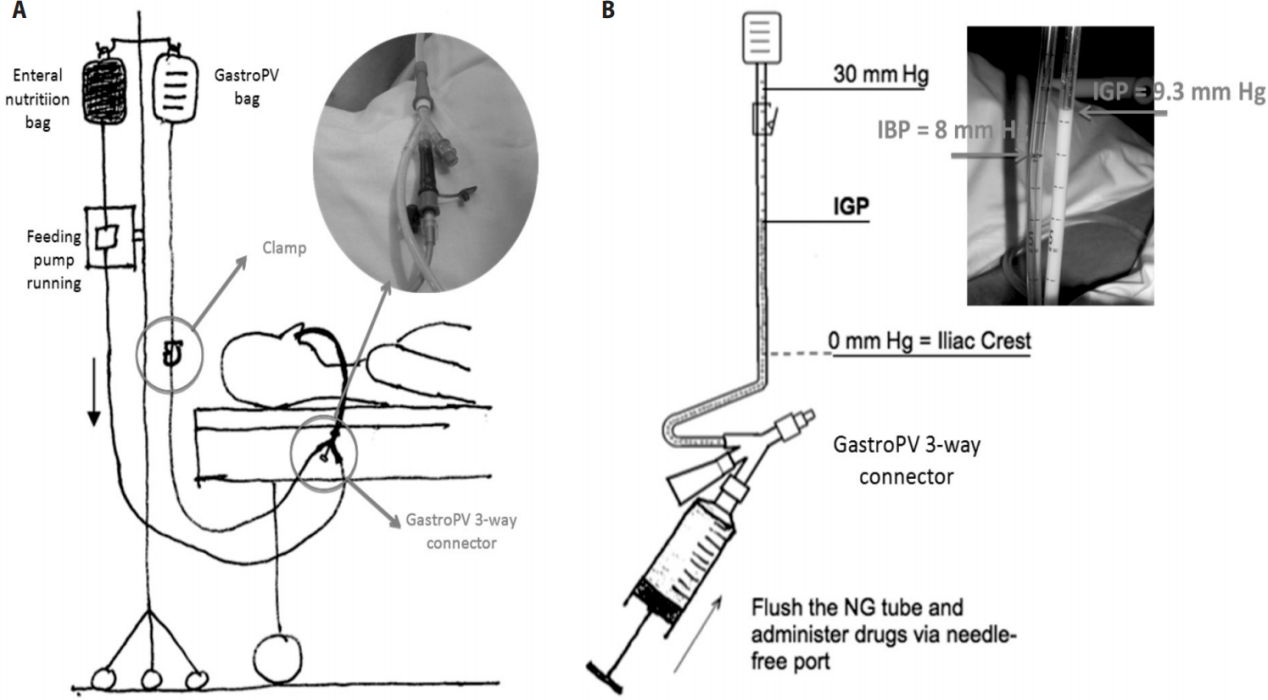


**Fig 12**. The GastroPV

**Panel A. Enteral feeding**

Preparations: 1) Stop the feeding pump 2) Insert Gastro PV between the NG tube and the feeding set; 3) Prime the tube with enteral feeding formula; 4) Start the enteral nutrition feeding pump at the desired speed

**Panel B. Intragastric pressure measurement**

To measure IAP via the GastroPV one must use the following steps: 1) Stop the feeding pump; 2) Place the bag on the bed; 3) Fill a syringe with 25mL H2O; 4) Inject 10 mL into the blue port; 5) Unclamp tube, and inject 15 mL; 6) Hold bag in vertical position, with 0 mm Hg at iliac crest; 7) Read IGP, then clamp tube; 8) Re-start feeding pump


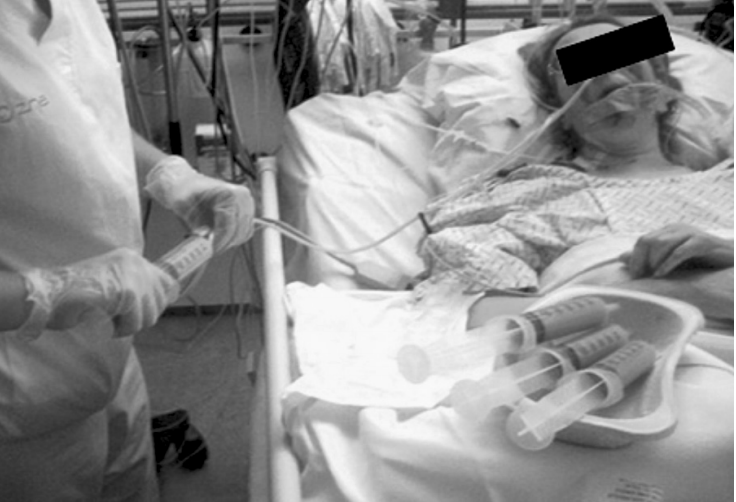


**Fig 13.** Classic gastric residual volume measurement

The enteral nutrition feeding pump is stopped and the tubing is disconnected. The gastric residual volume (GRV) is aspirated with a 60 mL syringe. Different syringes can be used. The total volume is calculated and the GRV is given back to the patient when < 300 mL (as per ICU protocol)


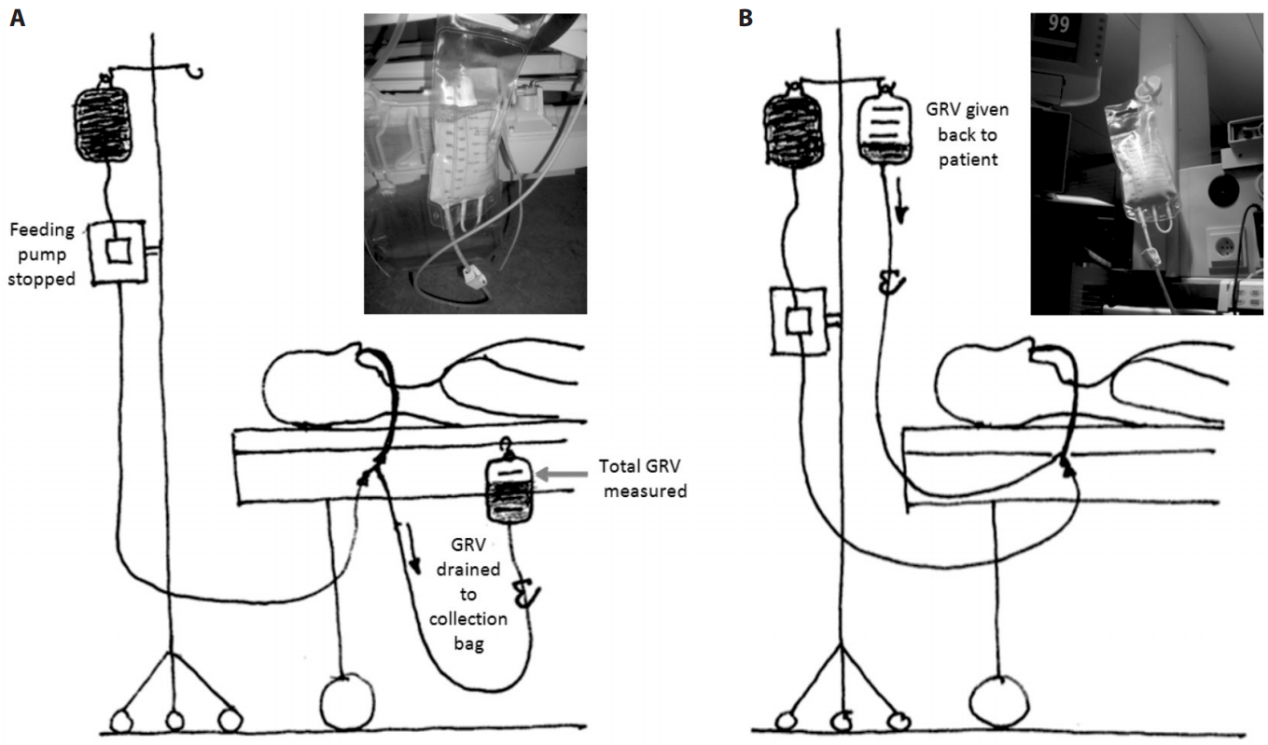


**Fig 14.** A new gastric residual volume measurement

**Panel A. GRV measurement**

To measure the Gastric Residual Volume, the feeding pump is stopped, the GRV collection bag is put on the ground or hung at the bedrail and the GRV is drained to the collection bag by gravity. If the bag does not fill spontaneously, or if bubbles appear in the tubing one can gently push the patient’s abdomen. Depending on the viscosity, it may take up to 15 minutes for the stomach to empty

**Panel B. Giving back GRV to patient**

To give back the GRV after measurement, the collection back is hung back and the GRV returns to the patient spontaneously by gravidity.

**11. Iacubovici et al. (2023)**

(1) Equipment

The Novel Continuous Bladder Pressure Monitor (Serenno Medical Ltd., Yokne’am Illit, Israel)

(2)Measurements

A urinary catheter was inserted after the induction of general anesthesia. The catheter was then serially connected to the study device, followed by a classic fluid–column manometer and a urine collection bag. If the initial urine volume was not suffificient to fifill the fluid manometer, up to 25 mL of saline was added to the system in a sterile fashion. Once the peritoneal cavity was insufflflated with carbon dioxide, the study device started to continuously record bladder pressures and a concealed envelope containing 5 random target pressures between 5 and 25 mmHg was opened. The laparoscopic insufflflator was set to reach and hold each pre-defifined pressure, and bladder pressures were recorded simultaneously from the two systems (namely the study device and the flfluid manometer). Each target pressure was kept for 30 s prior to recording to allow pressure stabilization. Once all 5 measurements were completed, the study device and flfluid manometer were disconnected, the bladder catheter was directly connected to the urine collection bag, and the surgical procedure was started. The study setup is illustrated in Figure 15. Each study measurement required about 1 min, and all measurements were completed in approximately 5 min. To reduce potential measurement bias, the study device recorded pressures based on an internal memory, so researchers were kept blinded to the device measurements. To assure paired recordings, the device was equipped with a button that was pressed when values were simultaneously recorded from the flfluid manometer. Each press of the button added a mark to the continuous pressure recordings, and only these corresponding values were considered for the analysis.

**
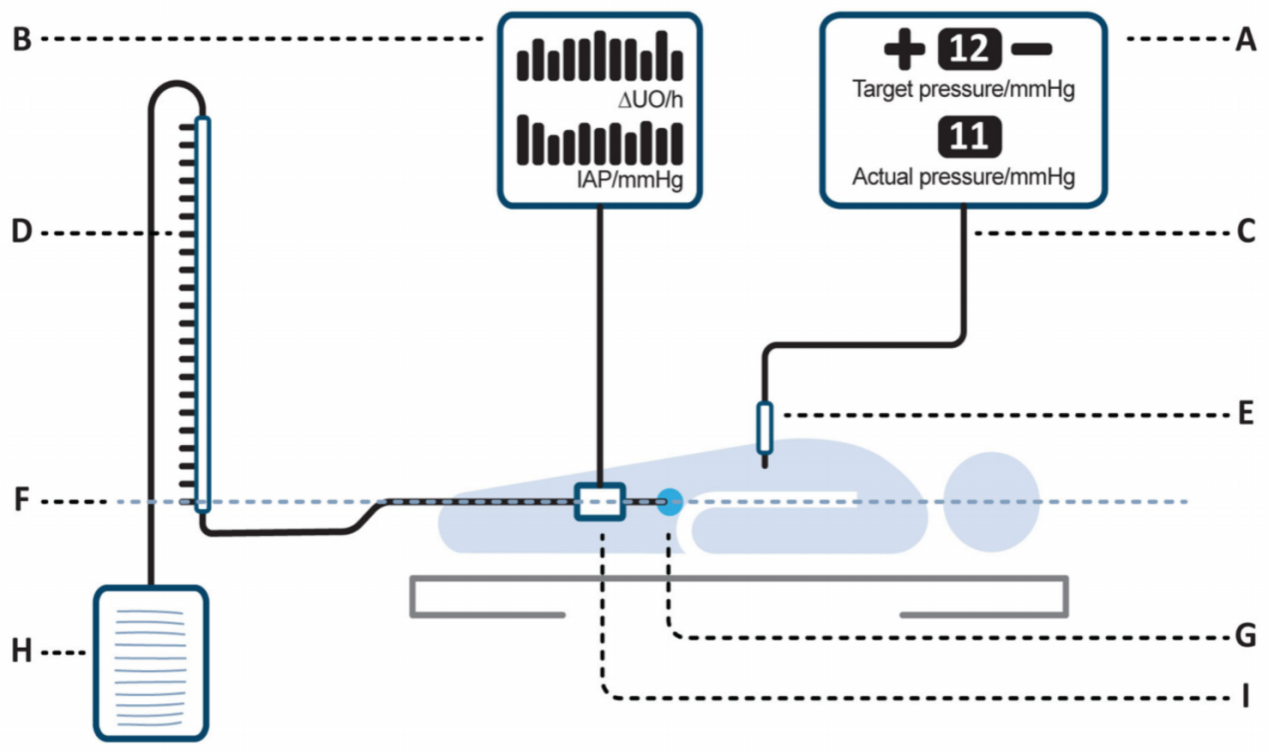
**

**Fig 15.** Study system design (not to scale). A—Insufflator—allows setting a pre-defined

1. **Camacho-Juarez et al. (2020)**

(1) Equipment

The novel transducer has a hermetic chamber and two valves to achieve pressure measurements.When the catheter connection valve (A) is plugged, the liquid or gas enters the transduction chamber (B), where the transducer sends air with a pressure proportional to the gas or liquid entering the measuring valve (C), where an electronic pressure measurement device displays the measured pressure in mmHg or cmH_2_O. The transducer chamber of the proposed pressure transducer was designed using SolidWorks computer-assisted design software (Waltham, MA, USA) and fabricated using the fused filament deposition printing technique which is a rapid and low-cost three-dimensional (3D) printing technique, based on depositing successive layers of melted printing material to conform a 3D object. The printing material was acrylonitrile butadiene styrene (ABS) plastic and the cost of the ABS plastic employed was < 2 dollars. The transducer chamber was divided into two subchambers separated by a thin Nylon membrane that allows the transduction of IAP to the sensor and avoids contamination of the instrument with the patient’s urine.


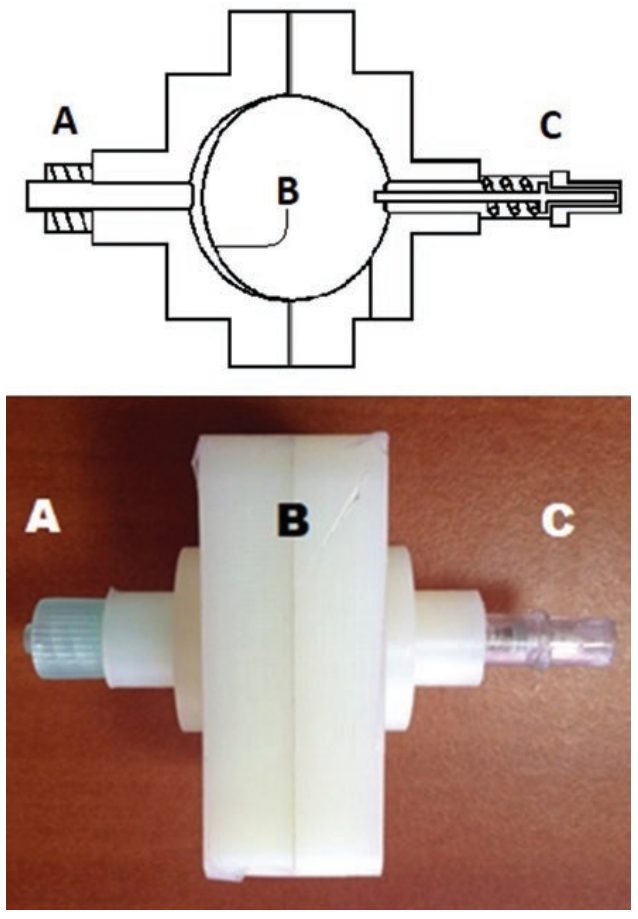


**Fig 16.** Schematic of the disposable transducer. A:connect valve to catheter. B:transduction chamber. C:air pressure measurement valve. Bottom: actual view of the disposable transducer.

(2) Measurements

① In vitro test simulating IAP measurements

As a simulator for the bladder, a serum bag filled with distilled water is used. By tightening the screws, a uniform pressure is applied to the bag in a random manner within the water range of 5.3 to 33.4cmH_2_0, located at the four corners of the mechanical press. Simultaneously use a disposable pressure sensor and a standard water column pressure gauge graded by cm water for measurement.


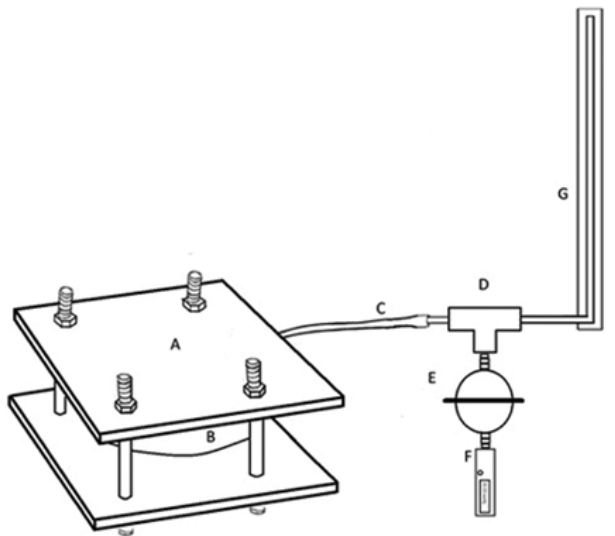


**Fig 17.** Schematic of in vitro simulated intra-abdominal pressure experiment. A: pressure B: water bag C: catheter D: three-way stopcock valve connector. E: mechanical pressure transducer with proximal and a distal valves. F: pressure measurer/calibrator. G: H_2_O water column graded manometer.

②Clinical trials

Clinical trial patients were measured using Kron's indirect method, which is the most commonly used method and is considered the standard for measuring IAP due to its low cost and high global acceptance. Kron's method is to place the welfare Foley catheter in the urethra until the patient's bladder. Three three-way valves (Luer locks) are placed in the urinary catheter, with one bag of physiological saline connected to the first Luer lock valve and one water column in centimeters connected to the second valve. Especially in this work, the third Luer lock valve was connected to a new pressure sensor device and IAP was continuously measured using two methods. Then, place the valve in the appropriate position to allow urine to flow into the bladder. When the urine stops flowing out, the urine drainage tube is blocked, allowing 25 ml of physiological saline to be injected into the bladder and measured with a water column. Data acquisition, inspiratory pause placed on the ventilator and sensor placed on the average axillary line or Pubic symphysis "reference 0 cm water" and physiological saline were allowed to connect the sensor and data through their respective Luer lock valves. Finally, place the Luer locking valve in the position for urine drainage.


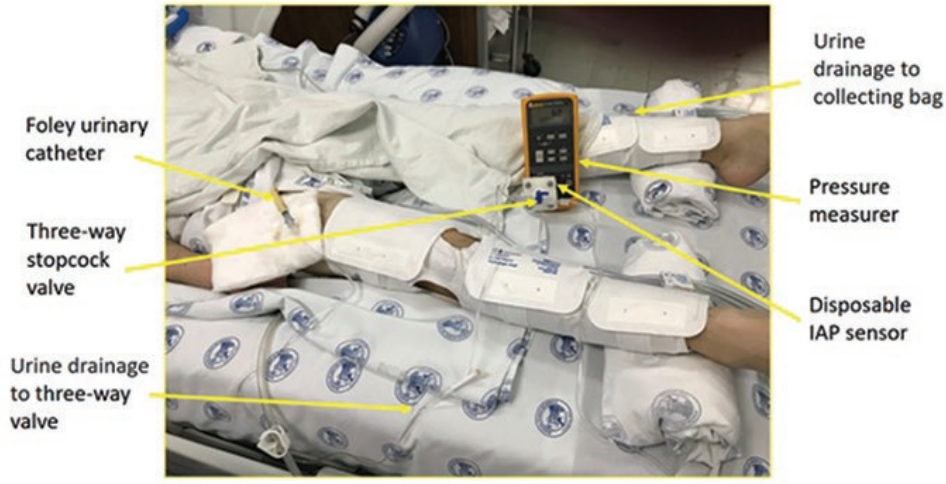


**Fig 18.** Measure of intra-abdominal pressure in a patient Using the proposed disposable transducer.

1. **David et al. (2021)**
2. Equipment

AbdoRF is a portable, relatively low-cost microwave refectometry system, designed to evaluate the refection coefcient of a patch antenna non-invasively attached to the abdominal wall. The hardware consists of the following parts:

①A generic microcontroller that commands and links between the hardware devices.

②A Voltage Controlled Oscillator (VCO) for radiofrequency wave generation in the range between 3.90 and

4.45 GHz.

③A wideband fexible patch antenna.

④A bidirectional RMS detector measures the power of the transmitted and refected wave through the antenna.

⑤A digital-to-analog converter and amplifers to control the VCO input.

⑥Analog-to-digital converters for sampling instantaneous RMS power values measured by (4), used for calculating S_11_


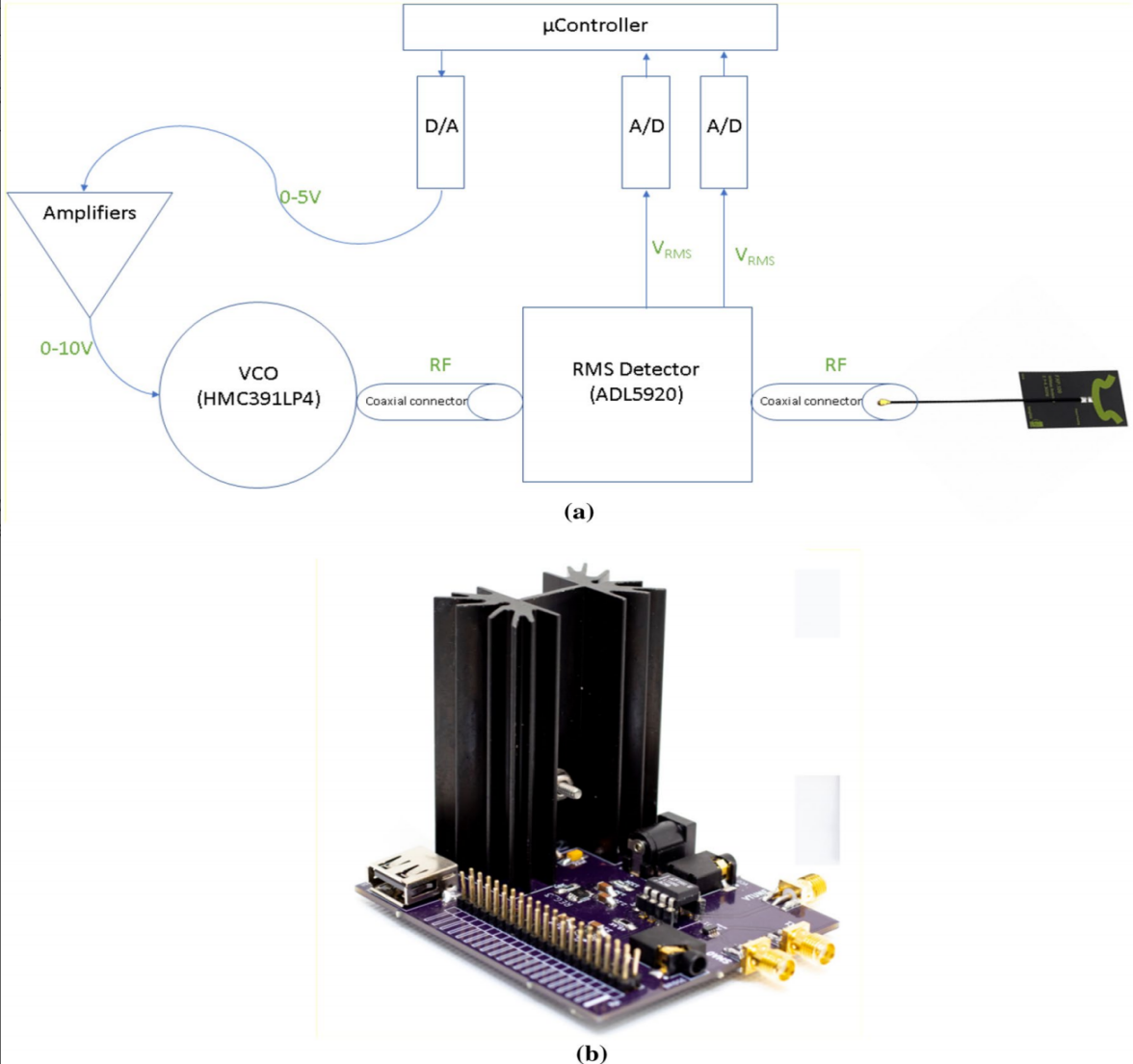


**Fig 19**. AbdoRF a system schematic diagram b picture of the device

(2) Measurements

Patients were in supine position; a trocar was inserted across the abdominal wall, reaching the intraperitoneal abdominal cavity for the laparoscopic pneumoperitoneum. Through the trocar, the abdominal cavity was infated using CO2 (infux of 0–30 l/min) to diferent pressures as needed for the surgical procedure. As recommended by WSACS (The Abdominal Compartment Society), the induced IAP was kept constant for about 60 s before performing the measurements, the measurements were performed at the end of expiration. Each refectometry measurement was performed five times for each IAP. Due to logistical constraints during the surgery, the antenna was placed over the left-inferior quarter of the abdominal wall, next to the linea alba as shown in Fig. 20.

**
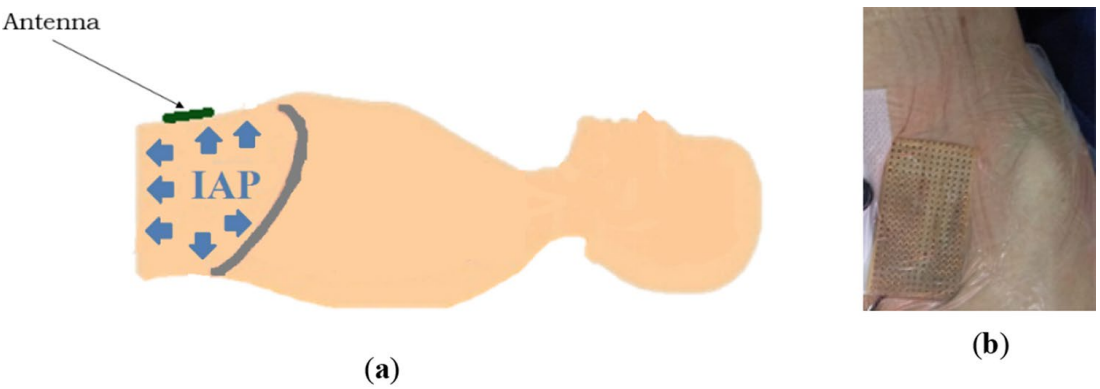
**

**Fig 20.** Antenna over the abdominal wall while the patient is in supine position: aschematic sagittal cut; b Patient A—the antenna is placed over the left-inferior quarter of the abdominal wal

1. **Gutting et al.（2023）**
2. Equipment

a three-lumen central venous catheter (CVC), a pressure transducer and an A/D converter on the patient-monitoring unit from GE (General Electrics).

(2) Measurements

**IVP:** based upon the modifified Kron technique, using a transurethral catheter according to WSACS recommendations (emptying the bladder, filling with 1 mL/kg of BW normal saline (min. 3 mL, max. 25 mL) under aseptc conditions, waiting for at least 2 min to allow equilibration) with the midaxillary level as the zero reference (clinical standard). IVP is transmitted from the end-open transurethral catheter through the continuous liquid column in the catheter lumen to an outside pressure transducer (Codan, Germany). Transurethral catheters for IVP measurement were sizewise adjusted for weight and age (Norta-Nelaton 6–16 Charriére (Ch.) diameter, BSNmedical

Company, Germany). For anatomical reasons, gastric tubes were used alternatively in small

neonates (Flocare pursoft tube, 5 Ch., Nutricia Medical Devices, The Netherlands).

**IGP:** was determined by air capsule-based measurement (Spiegelberg company, Germany) using a commercially available 9 French double-lumen nasogastric tube catheter with one lumen for continuous IGP measurement and another for regular feeding. The accurate intragastric or intravesical placement of the IGP and transurethral catheters was verifified by ultrasound at minimum once a day and, in addition, whenever the IGP or IVP measurements did not reveal respiratory undulations.

**FVP:** the femoral vein pressure (FVP) was measured via the flfluid-fifilled and flushed (1 mL/h), distal limb of a three-lumen central venous catheter (CVC) inserted into the femoral vein using the Seldinger technique (Vygon Multicath-3 Pädiatrie, Stolberg, Germany), with a pressure transducer and an A/D converter on the patient-monitoring unit from GE (General Electrics).

1. **Coleman et al. (2012)**
2. Equipment

A wireless intra-vaginal transducer: a microsensor, signal conditioner, microcontroller, power supply, and supporting wireless components into a gel filled elastomeric capsule.

**Capsule design:** the analog Gen1 IVT was packaged in the Rev1 capsule that was modeled after female hygienic devices and consisted of a cylindrical elastomeric capsule measuring 27.4 mm in

length, 12.7 mm in diameter and ending in a rounded terminus. To incorporate proposed

wireless components while preserving retention and performance characteristics, modifications to the original capsule design were needed. The final capsule design (Rev4) consisted of a large diameter base of 23.9 mm tapering to 14.7 mm diameter at the distal terminus with an overall length of 37.3 mm. Capsules were injection molded using machined aluminum molds with silicone elastomer (MED-4940, Nusil) cured at 150°C for 5 min.

**Microsensor package design and assembly :**space limitations of the capsule and size of electronic components required the electronic circuitry be separated into two electronic circuit boards. The insertable medical device (IMD) board side A (Fig. 21(a)) consisted of a pressure sensor (gauge-type microsensor— 3SC 2000 IT, Merit Sensor Systems), signal processor (ZMD31014GID1, ZMD

International Inc.) and four large solderable pads for attachment of the brass battery contacts. The battery contacts supplied power and ground from the coin cell battery while providing structural support to the electronics package. IMD side B (Fig. 21(b)) consisted of a MSP430 series microcontroller (MSP430F2132TRHBT, Texas Instruments) with 512 B onboard RAM for data buffering. Additionally, side B contained eight connection pads that served as the communication link between the IMD board and RF board through eight 34 gauge magnet wires (34 MAG, All Spectrum Electronics). The radio frequency (RF) board side A (Fig. 21(c)) consisted of eight connection pads for

communication with the IMD board and a Zarlink wireless chip (ZL70101LGD1, Zarlink).

Wireless communication was achieved by utilizing the ISM radio band 402–405 MHz

channel that is allocated for medical device communication by the FCC (wireless.fcc.gov).

Side B of the RF board (Fig. 21(d)) consisted of matching RF network, antenna, and four

connection pads for temporary attachment of communication wires for programming and

calibration. As with the IMD board, power to the RF board was supplied through four brass

contacts from the battery. A piezoresistive pressure die was secured to the prepopulated IMD electronics board with a small amount of UV-cure adhesive (3311, Loctite) to one corner of the die. The sensor die was microbonded to the IMD board mating pads using a manual wirebonder (West Bond 7476D-79) with aluminum 1% silicon 75 μm diameter bonding wire (CFW0014026,

California Fine Wire). The sensor die and bonded wires were sealed and reinforced using a

small amount of UV-cure alkoxy silicone (5248, Loctite). The battery holder provides a structural frame that secures the IMD, battery, and RF circuit board into an electronics package able to fit within the capsule. An assembly drawing of the electronics package is shown in Fig. 22. A knife plotter (Cutting Pro FC7000-75, Graphtec) was programmed to cut out an array of battery protectors from a 0.010″ polyester sheet as designed in a 2D computer-aided design CAD program (AutoCAD 2010, Autodesk). Each battery protector was separated from the sheet, folded around a 16 mm diameter aluminum rod and secured using UV-cure adhesive (3311, Loctite). Battery contacts were measured

and cut from .006 in. thick brass shim stock. A short circuit protector was made using .002 in. polyester along with a knife plotter and CAD software. Once cut, the short protector was placed over the negative terminal of the coin cell battery (CR1632, Panasonic) preventing the brass shim stock from simultaneously contacting both the positive and negative terminals. A contact protector was designed to prevent electronic components on the circuit boards from contacting the battery and was made using .010″ thick polyester sheet. Battery contacts were threaded though the precut slits of the battery protector securing the battery in the holder. The battery contacts were bent to accommodate both RF and IMD circuit boards in subsequent assembly steps. Contact protectors were placed at each end covering the brass contacts at both ends. Clear polyester shrink tubing .002″ thick (789200CST, Advanced Polymers) was cut to length and placed around the battery protector, heated, and secured in

place around the outside of the battery holder. A vent tube 0.027″ outside diameter 0.001″ wall thickness (Polyimide, Small Parts) was cut to length and threaded through the vent hole on the circuit board along with 0.2″ of exposed strain relief filament from the tether. UV-cure adhesive (3311, Loctite) was used to secure both the vent tube and strain relief to RF circuit board. The vent tube was then threaded through the hollow portion of the strain relief tether. The tether consisted of an extruded

silicone elastomer with incorporated nylon filament. A coaxial cable (SMA RG-178, Digikey) was soldered to the RF electronics board. A patch antenna (ZLE70101BADA, Zarlink) was then attached to the coaxial cable prior to use. Circuit boards were prepopulated on both sides with all components including the microbonded sensor die. Solderable magnet wire (34 AWG, All Spectrum Electronics) was cut to length and soldered into via holes on the IMD circuit board side B. The magnet wire

was threaded through the side wall between the battery and the battery protector and soldered to the corresponding pads on the RF circuit board side A. The battery contacts were folded making contact with the power pads located on IMD board side A and RF board side B and soldered. Once assembly of the electronics package was complete, four 32 gauge wires (32 AWG flex wire, Daburn) were soldered onto the programming pads on the RF board.

**Final transducer assembly** :wireless devices were assembled by first pre-filling elastomeric capsules with silicone gel (MED6350, Nusil) and degassing the gel in a vacuum at −22 inHg. The IMD board side A and RF board side B PCBs were primed using a silicone primer (MED1-161, Nusil) and

dried for 30 min at room temperature to improve the adhesion of the silicone gel and elastomer. The electronics package was then pressed into the capsule until no air bubbles were observed. The silicone gel and electronic package was cured in place at 70°C for 30 min. Silicone elastomer was used to pot the end of the capsule using an injection mold system. The devices were then allowed to cure at 150°C for 10 min. A cross-sectional view of the completed sensor design (absent elastomer potting and patch antenna) is shown in Fig. 23.

**Wireless prototype bench testing :**Programming and calibration of wireless Gen2 IVT prototypes was achieved through a fourwire JTAG connection using an interface board, MSP430 debugger (MSP-FETU430IF, Texas Instruments) and a computer. The IVT was sealed in a pressure chamber allowing the vent tube to be exposed to atmospheric pressures. Sensor output of three parameters:

atmospheric offset, gain, and temperature coefficient of offset (TCO) were needed for calibration. To obtain the atmospheric offset counts, the ZMD signal processor averaged pressure counts of the Wheatstone bridge for 5 s at a frequency of 30 Hz. Next, gain was obtained by pressurizing the vessel to 5 psi (350 cm H2O) using a NIST traceable reference transducer (Testcom, ER3000) at room temperature and averaging the pressure counts for 5s at 30 Hz. The TCO was calculated by calibrating the ZMD internal thermistor at room temperature and at 37°C at atmospheric pressure. The offset of the sensor was normalized by setting 0 psi to 8,192 counts and gain set to 16,383 counts at 5 psi (350 cm H2O). A pressure chamber was built to test the impulse and frequency response of the wireless

Gen2 IVT as shown in Fig. 24.


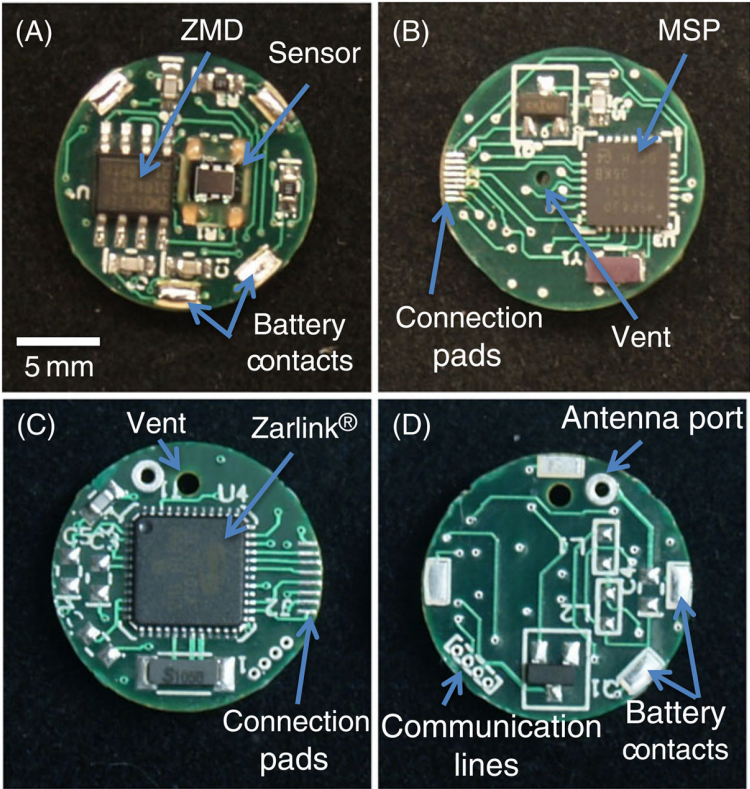


**Fig. 21.** Photograph of circuit boards and main features for wireless Gen2 circuitry. The IMD side A

(A) contains the piezoresistive pressure sensor, ZMD signal processor and battery contacts.

IMD side B (B) contains the MSP430 microcontroller and eight connection pads for linking

the IMD board to the RF board. RF side A (C) contains the Zarlink wireless chip and

connection pads. RF side B (D) contains four communication lines for programming and

calibration and battery contacts


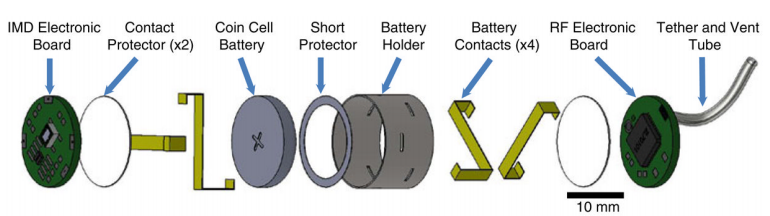


**Fig. 22.** Assembly drawing of electronic package. The battery is held in place by the four battery contacts. The contact protector at each end prevents the battery contacts from interfering with the IMD and RF circuit boards. Absent from this drawing are the shrink tubing, coaxial cable, and interboard communication wires


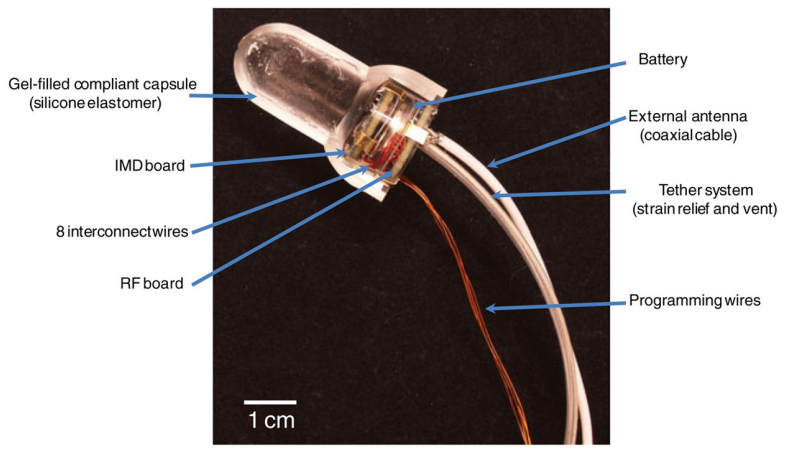


**Fig. 23.** Photograph displaying a cross section of the wireless intravaginal transducer. A coin cell

battery is sandwiched between the IMD and RF circuit boards. The clear battery holder

houses the electronics and battery in place supported by brass battery contacts. Four

programming wires allow for programing and calibration. These wires are removed after

calibration and prior to final device assembly. The tether is used for device retrieval and

serves as an atmospheric vent. An coaxial cable allows attachment of an external antenna


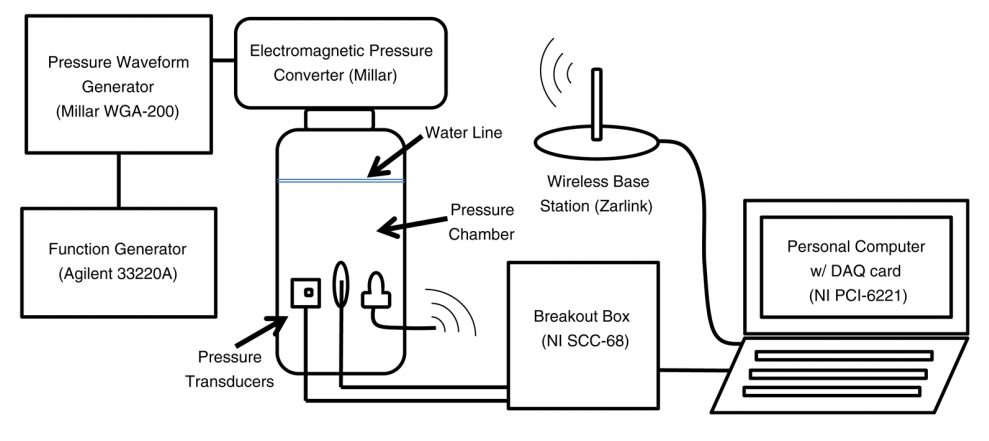


**Fig 24.** Dynamic response test setup. All three pressure transducers were sealed in the pressure

chamber. (from left to right: reference transducer, rectal balloon catheter and wireless Gen2

IVT)

(2) Measurements

A sterile wireless Gen2 IVT and standard rectal balloon catheter was placed in each study participant by a member of the clinical research team. Each participant performed a series of coughs followed by a Valsalva maneuver both in the seated and standing position. Rectal balloon IAP readings were recorded at 15 Hz using clinical cystometry software (Laborie Medical Technologies) under a standard bladder volume of 200 ml. Wireless IVT pressure measurements were taken at 30 Hz using the Zarlink ADK base station and custom laptop software. Results of each transducer were plotted and compared.

1. **Otto et al.（2010）**
2. Equipment

The tubing system and a Foley catheter.

(2) Measurements

Using Harrahill’s technique (UDM) to measure IAP, the tubing system itself served as the standpipe. To minimise the risk of urinary tract infection a T-piece (BARD® Covington, GA) was placed into the catheter tubing analogue according to Sugrue’s modiWcation. For measuring, the tubing system, Foley catheter, and bladder were Wrst Xushed with 50 ml sterile saline. This Xuid was completely drained leaving no air in situ before another 50 ml saline was injected serving as measurement volume. Using a ruler, pressure readings were obtained at the end of the expiration. The level of the symphysis always served as reference and readings in cmH2O were converted into mmHg by multiplying by 0.74 (Fig. 25). Following the measurement report, each IAP measurement consists of three comparison measurements. Each time, we began with IVM and went on with the Wrst UDM (UDM-1) and a second UDM (UDM-2). These comparison measurements were performed back-to-back within a short period of time.


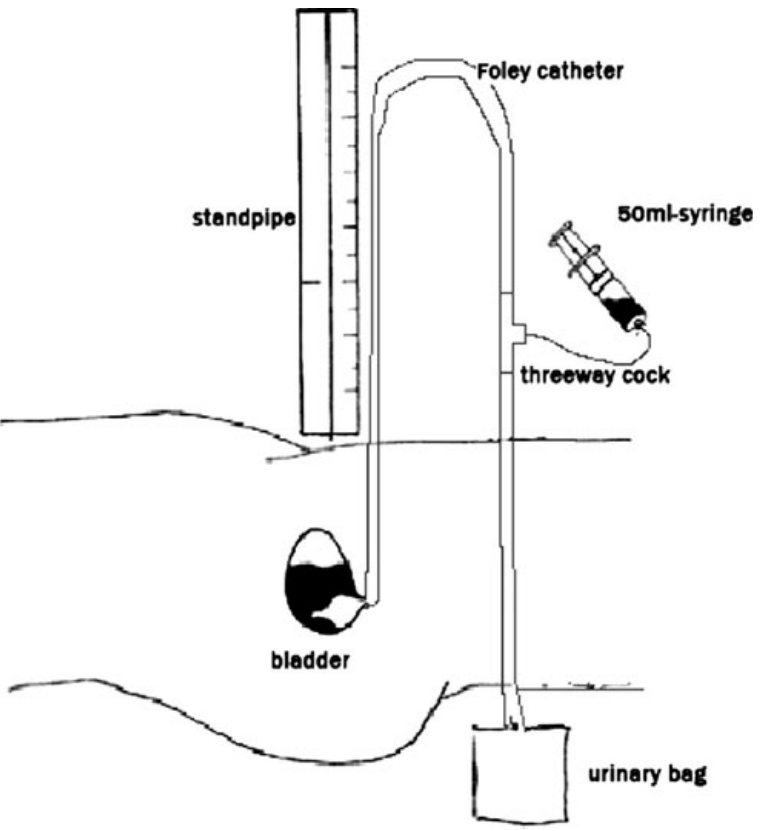


**Fig 25.** IAP measurement via Harrahill’s technique; measurement setup
