# Supplementary material for: The development, feasibility and credibility of intra-abdominal pressure measurement techniques: A scoping review
Source: PLoS One. 2024 Mar 21;19(3):e0297982. doi: 10.1371/journal.pone.0297982 (PMC10956852; doi:10.1371/journal.pone.0297982)
Supplement: S1 Table — (DOCX) [file pone.0297982.s003.docx]

**S1 Table. Characteristics of included studies**

| **Included study** | **Country** | **N** | **Number of measurements** | **Research objective** | **Research design** | **Participants** | **Measuring methods** | | **Results** | **Conclusion** |
| --- | --- | --- | --- | --- | --- | --- | --- | --- | --- | --- |
|  |  |  |  |  |  |  | Test group Control group | |  |  |
| Staelens  et al.  (2023) | Belgium | 9 | 84 | Comparing the Rectal IAP measurements (IAPrect) against gold standard intra-vesical IAP measurements | Prospective cohort study | Sedated and ventilated patients admitted to the ICU | Rectal IAP measurements: a rectal T-DOC 7Fr airflled balloon catheter (Laborie Medical Technologies, Mississauga, Canada) connected to a computer displaying the IAP (Audact Pro database version 7.11, Ellipse Andromeda, Urotex, The Netherlands) | Intra-vesical method: a Foley Manometer Low Volume (FMLV, Holtech, Medical, Charlottenlund, Denmark) | ①Bland-Altman analysis: 95% LoA=−19 ~ 2.9 mmHg  ②Correlation: p≥0.25, R^2^<0.6, Lin’s CCC<0.8  Bias=− 8.1 mmHg, Precision= 5.6 mmHg | 1. IAPrect is higher when compared to IAPves, 2. The two techniques cannot be used interchangeably 3. The IAP diferences and trend evolution after position change, or the application of an external abdominal pressure belt, are not similar to IAPves 4. Poor repeatability of IAPrect measurement |
| Iacubovici  et al.  (2023) | USA | 29 | 288 | Comparing IAP measurements using the novel monitor and a gold-standard foley manometer | Prospective cohort study | Adults having laparoscopic surgery and requiring urinary catheter intra-operatively | Intra-vesical method: the novel continuous bladder pressure monitor (Serenno Medical Ltd., Yokne’am Illit, Israel) | Intra-vesical method: a gold-standard fluid-column Foley manometer | r = 0.962, p＜0.01, R^2^ = 0.93  Bland-Altman analysis:95%LoA =–2.9~2.2 mmHg | 1. The study device and the gold-standard method in the   range of 5 and 25 mmHg have good consistency   1. The system introduces several potential advantages over existing IAP monitoring systems, including less user-dependence, a decrease in the nursing workload, potentially improved accuracy, continuous (rather than intermittent) monitoring with no interruption to urine flow, and the lack of sterility disruption |
| Gutting  et al.  (2023) | Germany | 39 | 2238 | Comparing the measurement agreement between trans-femoral venous pressure  (FVP) and intravesical pressure (IVP) and intragastric pressure (IGP) in children | Prospective  cohort study | PICU patients who needed an IAP measurement via IVP or IGP and additionally a femoral central venous catheter | Femoral venous pressure measurement: a pressure transducer and an A/D converter | Intra-vesical method: the modifed Kron technique  IGP: air capsule-based measurement (Spiegelberg company, Germany) | 1. FVP-IGP   r^2^ = 0.13, mean bias=−0.8±4.4 mmHg  Bland-Altman analysis:95%LoA = −9.6~8.0 mmHg, PE= 55%   1. FVP-IVP   r^2^ = 0.14, mean bias=0.5±4.2 mmHg  Bland-Altman analysis:95%LoA = −7.9~8.9 mmHg, PE= 51% | 1. The measurement agreement was extremely low between trans-femoral venous pressure and intravesical pressure/intragastric pressure 2. The clinical use of trans-femoral venous pressure in critically ill children should be strongly discouraged |
| See et al.  (2023) | Singapore | 21 | 74 | Feasibility analysis of a novel non-invasive ultrasonographic method for the measurement of IAP in ICU | Prospective cohort study | Adult ICU patients without underlying  pathology of the anterior abdomen, with a clinical suspicion of IAH, and with an existing indwelling urinary catheter attached to a pressure transducer | Non-invasive ultrasonographic method | Intra-vesical method | 1. IAPUS1 vs. IBP   Bland-Altman analysis: 95% LOA=-2.32 ~ 3.10 mmHg  CV=17.10%, PE=34.12%, r=0.91, ICC=92.7%   1. IAPUS2 vs. IBP   Bland-Altman analysis: 95% LOA=-2.35 ~ 3.58 mmHg  CV=19.20%, PE=38.37%, r=0.90, ICC=92.7%   1. IAPUS1 vs. IAPUS2   Bland-Altman analysis: 95% LOA=-0.99 ~ 1.44 mmHg  CV=7.70%, PE=15.33% | 1. The novel ultrasound-based IAP method displayed good correlation and agreement between IAP and IBP at levels up to 15 mmHg 2. IAP measurement by ultrasonographic evaluation in combination with external pressure is a practical, non-invasive alternative to the IBP method and can be used in patients who suffer from intra-pelvic masses or bladder pathologies |
| Kusar et al.  (2022) | Slovenia | 30 | 480 | Evaluating the feasibility of using patch-like transcutaneous sensors to estimate changes in IAP | Prospective cohort study | Patients early in the  postoperative course afer abdominal surgery | Abdominal wall tension:a transcutaneous sensors (MC-System, TMG-BMC, Ljubljana, Slovenia) | Intra-vesical method: an IAP measurement system (UnoMeter AbdoPressure, ConvaTec, UK) | ①Bland-Altman analysis:95% LoA=14.45~14.31mmHg  ②Correlation  a.Transcutaneously estimated IAP values: r = 0.0076, p < 0.0001  b.Subcutaneous tissue thickness: r = 0.069, p=0.0008 | (1)The sensors have the potential to be used to monitor IAP, but it has low sensitivity and poor consistency with the gold standard method  (2)The association between the subcutaneous thickness and tension changes on the surface and the IAP was statistically signifcant |
| Jacobson  et al.  (2022) | Canada | 27 | 121 | Evaluating the validity and reliability of a novel, non-invasive tool and method to measure IAP in vivo | Prospective cohort study | 14 living participants and 13 cadavers | A novel device：a novel tool to non-invasively characterize pressurized, physiological vessels | Intra-vesical method: a manometer technique  . | Cadavers  ①Bland-Altman analysis: 95% LoA=-10.4 ~ 6.2 mmHg  ②Correlation: r = 0.622，p <0.001  Living participants  Correlation: r = 0.889，p <0.001 | (1)The novel device proved to be reliable and valid, particularly when compared to published IAP values  (2)Errors exist in the system that may reduce device robustness in monitoring IAP fluctuations, including sensor noise , air leakage at the device seal, and misalignment of the device against the skin (orthogonal deviation)  (3) The dynamic feasibility of the device requires further study |
| Tang et al.  (2021) | China | 127 | 127 | Analyzing the correlation between AWT measurement and intravesical pressure (IVP) measurement | Prospective cohort study | Critically ill patients admitted to the ICU | Abdominal wall tension:a noninvasive multipoint abdominal wall tension measurement device (Chinese patent No.ZL 201510799207.4) | Intra-vesical method: Malbrain’s modified sterile IVP measurement device was used | Correlation: r = 0.706，p <0.05 | 1. AWT measurement method has a good correlation with IVP 2. Preoperative assessment of the AWT can be an effective tool for identifying patients at high risk of IAH 3. AWT can serve as a new method for the clinical diagnosis of abdominal infection |
| David et al.  (2021) | Israel | 5 | 25 | Describing the design of a refectometry system and its proof-of-concept trial | Prospective  cohort study | Patients admitted by the emergency department for laparoscopy surgery  due to acute abdominal pathologies | Microwave refectometry system | Using intraperitoneal inflation to achieve different IAP values | ①There was an inverse correlation between IAP and the refection coefcient  ②IAP has a strong dependence on the body mass index | 1. A better understanding of the dynamics in the changes of the AWTh (during intra-abdominal hypertension) was needed to improve further development of a microwave-based technique 2. Further research is still needed to optimize the sensitivity of the system; this includes optimal placement of the antenna, and redesign of the antenna itself to get larger variations of S_11_ for changes in IAP |
| Kaussen  et al.  (2021) | Germany | 97 | In vivo test: 5734  In vitro test:344 | Comparing ACM-IGP with IVP both in vivo and in vitro (water column) | Prospective cohort study | Children admitted to PICU with a transurethral catheter and need for a nasogastric tube | Intra-gastric method: air-capsule-based measurement (ACM-IGP, Spiegelberg company, Germany) | Intra-vesical method: the modifed Kron technique | 1. In vitro measurements   r^2^= 0.97, Bias = 0.8±0.8mmHg, PE= 20%  Bland-Altman analysis: 95% LoA=− 0.8 ~ 2.4 mmHg   1. In Vivo measurements   r^2^: 0.95, Bias 0.3±0.8 mmHg, PE=23%  Bland-Altman analysis: 95% LoA=−1.3 ~ 1.9 mmHg | 1. In a large PICU population with high IAH prevalence, ACM-IGP agreed favourably with IVP 2. More widespread usage of ACM-IGP may improve detection rates of ACS in critically ill children |
| Camacho-Juarez et al.  (2020) | [Mexico](https://fanyi.so.com/?src=onebox#Mexico) | 20 | In vitro tests: 159  In vivo tests: 40 | Validating the new novel disposable sensor by vitro and vivo tests | Prospective  cohort study | ICU patients and a simulator of the bladder | Intra-vesical method: A novel disposable sensor | Intra-vesical method: Modified Kron technique | 1. In vitro measurements   r = 0.99, p < 2.2 ×10^−16^, Bias=0.135 cmH_2_O  Bland-Altman analysis: 95%LoA = −0.82~1.09 cmH_2_O   1. In Vivo measurements   r = 0.973, p < 5.46 × 10^−13^, Bias=0.018 mmHg  Bland-Altman analysis:95%LoA = −3.30~3.64 mmHg | 1. The proposed disposable sensor seems promising for clinical applications 2. The advantage of this pressure measurement device is its low production cost and its portability in all acute care areas of healthcare centers, especially those without monitors available |
| Wagner  et al.  (2020) | Germany | 8 | 29 | A safety and feasibility study analyzing longitudinal IAP measurements in children who underwent renal transplantation | Retrospective cohort study | Children after renal  transplantation | Intra-vesical method: a hydrostatic manometer, the UnoMeter™ AbdoPressure™ IAP Monitoring System (Unomedical, ConvaTec™,) | / | ①Time expenditure for IAP measurement(mean ± SD [range]): 2.1 ± 0.4 [0.6-3.2] minutes  ②All urine cultures were negative | 1. No severe complications occurred during the IAP measurements 2. Analysis of longitudinal IAP measurements demonstrated that IAP measurement is safe and feasible in children recovering from renal transplantation in the PICU |
| Thangarasa  et al.  (2019) | Canada | 12 | 72 | Evaluating the reliability of handheld Stryker pressure monitor in peritoneal dialysis patients | Prospective cohort study | Patients having a peritoneal dialysis  catheter inserted via laparoscopic surgery | Direct measurement: a handheld Stryker intracompartmental pressure monitor connected to the peritoneal dialysis catheter | Insufflator pressures: the insufflator (Olympus, USA) pressure was altered to 15, 10, and 5 mmHg | 15mmHg: ①Bland-Altman analysis: 95% LoA=7.9 mmHg  ②Correlation: ICCs = 0.634  10mmHg: ①Bland-Altman analysis: 95% LoA=12.2 mmHg  ②Correlation: ICCs = 0.015  5mmHg: ①Bland-Altman analysis: 95% LoA=10.3 mmHg  ②Correlation: ICCs = 0.212 | The STIC pressures were not a reliable estimate of the insufflator pressures especially at lower pressures |
| Deindl  et al.  (2019) | Germany | 23 | 425 | A safety and feasibility study analyzing longitudinal IAP measurements in children who underwent liver transplantation | Retrospective cohort study | Children after liver  transplantation | Intra-vesical method: a hydrostatic manometer, the UnoMeter™ AbdoPressure™ IAP Monitoring System (Unomedical, ConvaTec™,) connected to a Foley catheter | / | ①Time expenditure for IAP measurement(mean±SD [range]): 1.9 ± 0.4 (0.5‐3.2) minutes  ②Agitation had a significant impact on IAP: estimate: 9.3 mmHg, CI: 6.72‐11.97, P < .01  ③All urine cultures were negative | 1. The safety and low time expenditure associated with IAP measurement could be included easily into standard nursing procedures for these patients 2. Agitation was strongly associated with elevated IAP and impaired vascular flow |
| Al-Abassi  et al.  (2018) | United Arab Emirates | 21 | 469 | Comparing urinary bladder pressure (UBP) with IAP | Prospective cohort study | Patients undergoing laparoscopic cholecystectomy under general anesthesia | Bladder pressure (UBP) : DantecMenuet equipment (M 247) and fluid-filled lines with external pressure transducers | IAP:laparoscopy CO2 insufflator | 1. IAP= 0–22 mmHg   ①BlandAltman analysis:95% LoA=−2.83 ~ 2.64 mmHg  ②Correlation: r = 0.966, p < 0.0001   1. IAP > 12 mmHg   ①BlandAltman analysis:95% LoA=−0.62 ~ 0.05 mmHg  ②Correlation: r = 0.940, p < 0.0001 | Intra-vesical measurement can reliably estimates IAP in patients placed in supine position, but may be less reliable while measurements for pressures higher than 12 mmHg |
| Niederauer  et al.  (2017) | USA | 429 | 410 | Design, development, testing and preliminary clinical use of the novel intravaginal pressure transducer | Prospective cohort study | Women at eight weeks and one year postpartum | Vaginal IAP measurements: the novel intra-vaginal pressure transducer | / | 1. The wired sensors showed high linearity during calibration 2. There was not a significant change in thermal offset from room to body temperature of the sensor when the support ring was changed from aluminum | 1. The novel intravaginal pressure transducer is easier to use, less costly, and more reliable than previous designs, while maintaining accuracy, integrity, and quality of data 2. The novel intravaginal pressure transducer can be used to collect data for the Motherhood and Pelvic Health (MAP) study to examine the role of IAP on pelvic floor disorders |
| Howard  et al.  (2016) | Australia | 11 | 106 | Comparing IAP measurements via the bladder with femoral venous pressure (FVP) at baseline and with artificially increased IAP ≥20 mmHg | Prospective cohort study | ICU patients with a pre-existing femoral venous catheter and indwelling bladder cathete | Femoral venous pressure measurement: an engineered transducer device (Fremantle Hospital Medical Equipment Department, Fremantle, WA) | Intra-vesical method: the modified Kron technique using an AbViser 300 or 611-kit (Convatec Medical, Greensboro, NC, USA, formerly provided by WolfeTory Medical, Salt Lake City, UT, USA) | (1) 0 kg of weight  Bias= 3.2 mmHg , Precision= 3.63 mmHg  Bland-Altman analysis:95% LoA= -4.1 ~ 10.4 mmHg  (2) 5 kg of weight  Bias= 2.5 mmHg, Precision= 3.92 mmHg  Bland-Altman analysis:95% LoA= -5.4 ~ 10.3 mmHg  (3) 10 kg of weight  Bias= 2.5 mmHg, Precision= 2.26 mmHg  Bland-Altman analysis:95% LoA= -2.1 ~ 7.0 mmHg | 1. FVP cannot be recommended as a surrogate measure for IAP measurement via the bladder even at IAP >20 mmHg 2. FVP >17 mmHg was a predictor of IAH and may be used as a continuous screening tool if a femoral venous catheter has already been placed for venous access |
| Bunnell  et al.  (2015) | USA | 30 | 90 | To evaluate the reliability of peak inspiratory pressure (PIP), plateau pressure (Pplat), and mean airway pressure (Paw) as surrogate estimates of IAP during abdominal closure | Prospective cohort study | Mechanically  ventilated surgical/trauma patients with risk factors for IAH/ACS | Airway Pressures | Intra-vesical method:IAP monitoring system (AbViser IAP Monitoring System and a modified Harrahill technique | 1. PIP VS IAP   ①Bland-Altman analysis:95% LoA=0.6 ~38.0mmHg  ②Correlation:r=o.223   1. Pplat VS IAP   ①Bland-Altman analysis:95% LoA= −2.5 ~ 24.8 mmHg  ②Correlation: r= o.417   1. Paw VS IAP   ①Bland-Altman analysis:95% LoA=−7.9 ~ 11.8 mmHg  ②Correlation: r=o.388 | 1. Airway pressures do not accurately reflect IAP 2. Paw most closely approximates IAP although with marked variability |
| Chen et al.  (2015) | China | 51 | 306 | Analyzing the correlation  between AWT and urinary bladder pressure (UBP) | Prospective cohort study | ICU patients | Abdominal wall tension: a self-made AWT measurement device including one thrust meter (Aidebao Instruments Co. Ltd., Zhejiang, China) and one self-made device | Intra-vesical method: the AbViserAutoValve device (Wolfe Tory Medical, Inc., Salt Lake City, UT,USA; Figure 2) connected to a Fleischl catheter and an ICU general pressure sensor | Correlation: r = 0.986，p <0.01 | 1. It was feasible to use AWT to monitor IAP. The AWT method is very simple, fast, and accurate 2. To maintain the unity and stability of the AWT method, supine position during late expiration when using AWT to monitor IAP was recommend 3. The AWT measurement device should be standardized |
| Van et al.  (2014) | Canada | 37 | IVP:270  GRV: 292 | Validating a novel method combining measurement of Gastric residual volume (GRV) and estimation of IAP via intra-gastric pressure (IGP). | Multicentre retrospective cohort study | Mechanically ventilated  ICU patients | Intra-gastric method: A new device, inserted in between the nasogastric probe and the enteral nutrition feeding pump and tubing | Intra-vesical method: FoleyManometer (Holtech Medical, Charlottenlund, Denmark) | 1. GRV VS IBP   R^2^ = 0.51, Bias== 0.8 mmHg, Precision = 2.7 mmHg  Bland-Altman analysis: 95% LoA=−4.5~ 6.1 mmHg   1. GRVprototype VS GRVclassic   R^2^ = 0.89, Bias = -0.8 mL, Precision = 52.3 mL  Bland-Altman analysis:95%LoA =–103 ~ 102 mL | 1. GRV can be measured with the new device 2. The novel method reduces the nursing manipulations to measure the GRV and allows more frequent GRV measurements to anticipate possible GRV increases 3. Measurement of IGP does not carry a potential risk for urinary tract infections 4. The novel method is cost-effective, in particular for those cases with large amounts of GRV |
| Kruger  et al.  (2013) | New Zealand | 14 | 756 | Develop and test a novel, wireless intra-vaginal pressure sensor (IVPS) to quantify IAP changes across a range of well-defined activities | Prospective cohort study | Asymptomatic women without prolapse | Vaginal IAP measurements: a wireless intra-vaginal pressure sensor | / | Correlation between cycles across all variables:  r >0.935 (mean)  r>0.964 (amplitude) | 1. All women found the Vaginal IAP measurements comfortable and easy to insert 2. The device has shown excellent repeatability across a range of activities |
| Castañón-González et al.  (2013) | [Mexico](https://fanyi.so.com/?src=onebox#Mexico) | 5 | 50 | Validating a device and technique  developed to measure IAP (an innovation of the  Foley urinary catheter named IAP catheter | Prospective cohort study | Adults having laparoscopic surgery | Intra-vesical method: an innovation of the Foley urinary catheter named IAP catheter | Graded manometer  Direct measurement  Kron method | ①IAP catheter VS Graded manometer  r = 0.99 Bland-Altman analysis:95% LoA=0.039 ~ 0.092 mmHg  ②IAP catheter VS Direct measurement  r = 0.93 Bland-Altman analysis:95% LoA=−1.46 ~ 1.83 mmHg  ③IAP catheter VS Kron Method  r = 0.81 Bland-Altman analysis:95% LoA=−2.12 ~ 1.3 mmHg | The IAP catheter device is a safe and reliable instrument to measure intra-abdominal pressure |
| Hsu et al.  (2012) | USA | 16 | / | A criterion validity study was conducted among women undergoing urodynamic testing to determine the accuracy of the Wireless IAP Transducer prototypes when compared to accepted clinical standards | Prospective cohort study | Women | Vaginal IAP measurements：the wireless intra-vaginal pressure transducer | Rectal IAP measurements: the rectal balloon catheter | r=0.97 | 1. The wireless intra-vaginal pressure transducer comfortable to wear and easily retained during physical activity 2. IAP measurements from the fwireless intra-vaginal pressure transducer compared favorably to standard urodynamic transducers |
| Coleman  et al.  (2012) | USA | 7 | / | Developing a novel intra-vaginal pressure transducer continuously monitor IAP | Prospective cohort study | Women volunteers undergoing standard urodynamic testing | Vaginal IAP measurements：wireless Gen2 devices capsules | Rectal IAP measurements: a conventional urodynamics balloon catheter placed in the rectum | Pressure measurements made by the wireless Gen2 IVT were consistent with rectal catheter data in both coughs and Valsalva maneuvers | 1. Wireless technology allows users free mobility while providing the clinician accurate IAP monitoring during daily activities 2. There is the ability of the wireless Gen2 IVT to accurately and reliably measure IAP when compared to the reference and rectal balloon catheters |
| Desie et al.  (2012) | Belgium | 1097 | / | Determine whether IAP monitoring using the FoleyManometer increases the risk of urinary tract infection. | Retrospective cohort study | ICU patients | Intra-vesical method:  ①a modified homemade technique,  ②a FoleyManometer with 35 ml reservoir,  ③a FoleyManometer low volume (FoleyManometerLV) with less than 10 ml priming volume | / | ①Crude and adjusted UTI rates per 1,000 catheter days (CD) were on average 16.1 and 12.8/ 1,000 CD, respectively, and were not significantly different between the four time periods  ②The number of positive UC as a ratio to the number of UC taken decreased in period 4 as compared to the control group from 12.5% to 9% (p = 0.007) | Intrabladder pressure monitoring as estimate for IAP either via a closed transducer technique or the closed FoleyManometer technique seems safe and does not alter the risk of UTI in critically ill patients |
| De Keulenaer  et al.  (2011) | Australia | 149 | 1732 | To investigate the correlation between femoral venous pressure measurement and intrabladder pressure measurement | Multicenter prospective cohort study | ICU patients had a femoral venous catheter and an indwelling bladder catheter already in place | Femoral venous pressure measurements: a FoleyManometer | Intra-vesical method:a FoleyManometer | 1. IAP≥20 mmHg   Bias= 0.7 mmHg, Precision= 2.0 mmHg  Bland-Altman analysis:95% LoA=−3.0 ~ 4.6 mmHg   1. IAP≥12 mmHg   Bias=0.4 mmHg, Precision=3.9 mmHg  Bland-Altman analysis:95% LoA: −8.1 ~ 7.3 mmHg | 1. FVP cannot be used as a surrogate measure of IAP unless IAP is above 20 mmHg 2. The use of FVP as a continuous measure of IAP is important in the prevention of early ACS |
| Chiumello  et al.  (2011) | Italy | 24 | 96 | Compare the esophageal pressure (Pes) and intragastric pressure  (IGP) measured using this new device with those obtained with a standard balloon catheter taken as gold standard | Prospective cohort study | Intubated patients in ICU requiring ventilator support | Intra-gastric method: the new nasogastric polyfunctional catheter (Nutrivent, Sidam, Mirandola, Italy) | Pes and IGP: a standard balloon catheter (Smart Cath, Viasys, Palm Springs, USA) | 1. Esophageal pressure (Pes)   Bias= -0.25 cmH2O  Bland-Altman analysis: 95% LOA= -2.65 ~ 2.15 cmH2O   1. Intragastric pressure (IGP)   Bias= -0.45 cmH2O  Bland-Altman analysis: 95% LOA= -2.85 ~ 1.95 cmH2O | 1. The new polyfunctional catheter showed a clinically acceptable validity in recording esophageal and intragastric pressure |
| Van Ramshorst  et al.  (2011) | Netherlands | 14corpses  42students | 2010 | To assesse the relation between IAP and abdominal wall tension (AWT) in vitro and in vivo | Prospective cohort study | Human corpses (up to 1 wk post mortem and adequately cooled) and healthy students | Abdominal wall tension:a built-in force and distance sensor, attached to a handheld personal digital assistant | Insufflator pressures | In vivo measurements  ①AWT was highest at points 1 and 2  ②AWT was on average 31% higher in men compared to women  ③Mean CV of repeated measurements was 14%  ④BMI did not influence AWT  ⑤AWT levels increased significantly from late expiration to inspiration to Valsalva’s manoeuvre ( P <0.001)  In vitro measurements  ①all points showed significant correlations between IAP and AWT  ②Mean slopes were greatest at points across the epigastric region (points 1–3) | 1. All points showed significant correlations between IAP and AWT (P < 0.001), but the repeatibility of measurements was low 2. Points 1 and 2 were found most suitable for detection of changes in IAP and AWT 3. Further longitudinal clinical studies are needed to assess usefulness of AWT measurements for monitoring of IAP |
| Otto et al.  (2010) | Germany | 43 | 222 | Evaluate the correlation, reproducibility and  effctiveness of Harrahill’s technique | Prospective cohort study | Patients undergoing elective standard abdominal intervention with laparotomy | Intra-vesical method: Harrahill’s (UDM) technique | Intra-vesical method: Kron’s (IVM) technique | 1. Correlation:   r= 0.97, Mean difference= -0.2±0.9 mmHg  Bland-Altman analysis: 95% LOA= -1.7~ 2.0 mmHg   1. Reproducibility:   r= 0.97, Mean difference= -0.1±1.1 mmHg  Bland-Altman analysis: 95% LOA= -2.3~ 2.2 mmHg   1. Time consumption (UDM 215 s vs IVM 361 s) and costs (UDM 1.89 D vs IVM 4.27 D) for the first measurement | 1. Harrahill’s technique has good correlation and high reproducibility of IAP measurement compared to the gold standard Korn method 2. Harrahill’s technique is a suitable method for quick and simple screening test for intra-abdominal hypertension, especially after repair of giant incisional hernias 3. Harrahill’s technique is simple as well as economic in both time and costs for the frst measurement |
| Vallee  et al.  (2010) | France | 78 | 270 | To demonstrate the possibility of accurately measuring IAP by using a common urine drainage bag (U-Tube) as a hydrostatic column of measurement | Prospective cohort study | Patients newly admitted in ICU | Intra-vesical method: two U-Tube  ①U-Tube method I, where *h* (in cm) alone assesses IAP (in cm H2O)  ②U-Tube method II, integrating BUV according to a basic biomechanical model of  bladder wall compliance | Intra-vesical method: a Foley-catheter, a 3-stopcock ramp and a urine drainage system | 1. U-Tube method II   R^2^ = 0.901, P＜0.0001  Mean bias=-1.0±0.1 mmHg  Bland-Altman analysis:95% LOA= -3.4~ 1.4 mmHg   1. U-Tube method I   R^2^ = 0.682, P＜0.0001 | 1. By integrating urine column height and BUV in the measurement method, it may be conceivable to screen IAH at the bedside via a U-Tube in ICU 2. Bladder wall compliance should be estimated to avoid the emergence of false-positive subjects due to the possible occurrence of bladder wall compliance alteration before or during the ICU stay |
| Van der Steeg et al.  (2009) | Netherlands | 15 | 105 | To evaluate the effificacy of the urine column (UC) measurement compared to the intra-vesicular pressure (IVP) measurement as an estimation of IAP in patients with IAP up to 30 mmHg | Prospective cohort study | Patients undergoing a laparoscopic cholecystectomy | Intra-vesical method:urine column (UC) measurement | Intra-vesical method | 1. 50 ml saline priming volume   r=0.91, P＜0.0001  Bland-Altman analysis:95% LOA=-7.21 ~ 5.85 mmHg   1. 100 ml saline priming volume   r=0.87, P＜0.0001  Bland-Altman analysis:95% LOA= -7.41 ~ 9.87 mmHg | 1. UC measurement shows signifificant correlation to IVP   measurement as an estimation of the IAP   1. Further study needs to be done to conclude whether UC measurement is a reliable clinical alternative to IVP measurement |
| Van Waes  et al.  (2009) | Netherlands | 25 | 250 | To evaluate the use of a single-lumen central venous catheter  to measure IBP as a  representative of the true IAP | Prospective cohort study | Surgical patients admitted to ICU | Direct measurement: a single-lumen central venous catheter (CVC) placed through the abdominal wall into the abdominal cavity to continuously | Intra-vesical method: the modifed Kron technique | Mean difference:1.6±2.7mmHg.  ICC= 0.82 | 1. The continuous and direct IAP measurement using a single-lumen central venous catheter is accurate and making it easier for the nursing staff to be informed of the IAP 2. Patients who have not undergone laparotomy but are at risk of ACS (e.g.,trauma, peritonitis) would best be monitored with the continuous IBP technique |
| Otto et al.  (2009) | Germany | 20 | 52 | To assess Piezoresistive pressure measurement technique (PRM)  regarding complications, reliability and agreement with IVP in patients  undergoing elective abdominal surgery | Prospective cohort study | Patients scheduled  for elective abdominal surgery | Direct measurement: via piezoresistive pressure measurement was done with two different probes: Coach®-system (CPRM, MIPM, Mammendorf, Germany) and Accurate++®-probe (APRM, MIPM, Mammendorf, Germany,) | Intra-vesical method | Bland-Altman analysis: 95% LOA=-5.5 ~ 5.6 mmHg  Mean difference= 0.1±2.8 mmHg | 1. Although results of Accurate++® were comparable to IVP, the device might be too fragile for IAP measurements in the clinical setting 2. There were no signs of probe related organ lesion or surgical site infection |
| Becker et al.  (2009) | Germany | 10 | 180 | To evaluate a new commercially available system for the intra-gastric measurement of IAP | Prospective cohort study | Patients with cirrhosis  and tense ascites scheduled for para  centesis | Continuous intra-gastric measurement (CiMon): Pulsion Medical Systems, Munich,  Germany | Direct measurement: through the  paracentesis catheter using a scaled  measurement gauge | R^2^= 0.38 Bias=-4.9 mmHg  Bland-Altman analysis: 95% LOA=-6.8 ~ 6.8 mmHg | 1. CiMon device for intra-gastric measurement of IAP underestimates IAP when compared with direct intra-peritoneal measurements in ascitic patients and that measurements between the two methods do not correlate well. 2. This system cannot be regarded as a reliable substitute for standard techniques of IAP measurement |
| Zengerink  et al.  (2008) | Canada | 79 | 300 | Evaluating a new technique  for continuous monitoring of intra-abdominal pressure (CIAP) using a standard threeway bladder catheter in a diverse group of ICU patients | Prospective cohort study | ICU patients | Continuous monitoring of intra-abdominal  pressure (CIAP) : using a standard three  way bladder catheter | Intermittent IAP (IIAP) measurements: using a modification of the original method Kron et al., as described by Cheatham and Safcsak | Bland-Altman analysis: 95% LOA=-4.12 ~ 4.0 mmHg  ①When IIAP ＜ 20 mmHg  Bland-Altman analysis: 95% LOA=-3.7 ~ 3.7 mmHg  ②When IIAP ≥ 20 mmHg  Bland-Altman analysis: 95% LOA=-5.8 ~ 5.1 mmHg | 1. CIAP is an accurate and simple means of measuring IAP when compared with the current standardized method 2. CIAP was sensitive for detecting slightly elevated IAP (>11 mm Hg) but is less sensitive for distinguishing between higher grades of IAH (e.g., IAP >20 mm Hg or 25 mm Hg) 3. Elevated CIAP measurements should be confirmed with IIAP measurements if accurate grading is required until further validation and experience is obtained |
| Otto et al.  (2008) | Germany | 30 | 336 | To assess direct IAP measurement using an aircapsule method (ACM) regarding complications risks and agreement with IVP in patients undergoing abdominal surgery | Prospective cohort study | Patients undergoing elective colonic,  hepatic, pancreatic and esophageal resection | Direct measurement: an air-capsule technique(Spiegelberg®-System, Probe 3, Hamburg, Germany) for the direct measurement of IAP | Intra-vesical method | Bland-Altman analysis: 95% LOA=-4.1 ~ 5.1 mmHg  Mean difference= 0.4±2.2 mmHg  r=0.69 | 1. Using ACM, direct IAP measurement is feasible and uncomplicated 2. Associated with relatively low pressure ranges (<17 mmHg), results are comparable to bladder pressure measurement. |
| Pracca  et al.  (2007) | Italy | 11 | 184 | To describe a direct IAP measurement technique using a solid microsensor comparing its values with the ones simultaneously obtained by means of Kron’s technique | Prospective cohort study | ICU patients considered irreversibly ill, without therapeutic possibilities | Direct measurement: using a solid microsensor | Intra-vesical method: Kron’s technique | Mean difference= 0.286±0.938 mmHg  r= 0.98  Bland-Altman analysis:95% LOA= between 1.87 mmHg | 1. Codman sensor, allows continuous monitoring of IAP without urinary tract manipulation 2. Due to the cost direct IAP measurement should be reserved for selected critical patients where standard techniques are contraindicated or can be inaccurate 3. No complications with direct IAP measurements were found |
| Kimball  et al.  (2007) | USA | 18patients  89 nurses | 212 | To evaluate theintraobserver and interobserver variability of bladder pressure measurements | Prospective cohort study | ICU patients | Intra-vesical method: IAP monitoring system (AbViser, Wolfe Tory Medical, Salt Lake City, UT, USA) connected with the ICU standard pressure transducer | / | 1. Intra-observer   r=0.934, p < 0.001  Bland-Altman analysis:95% LOA=-2.938 ~ 4.078 mmHg   1. Inter-observer   r=0.950, p < 0.001  Bland-Altman analysis:95% LOA=-3.069 ~ 3.069 mmHg | 1. IAP can be accurately and reliably measured in critically ill patients by utilizing a standardized measurement device combined with a standardized clinical protocol 2. Obtain frequent data points to establish a trend rather than relying on a single measurement to make important management decisions is needed |
| De Waele  et al.  (2007) | Belgium | 7 | 312 | To compare IAP measurements obtained from an intragastric Compliance catheter with the pressure measured directly  in the abdominal cavity | Prospective cohort study | Patients undergoing elective  laparoscopic cholecystectomy | Intra-gastric measurement: using a compliance catheter (International Medical Systems, Zutphen, The Netherlands) | Direct measurement: measured directly in the abdominal cavity | Mean difference: 0.12 ± 0.70 mmHg (95% CI 0.01–0.23) | IAP measured using an intragastric Compliance catheter reliably reflects the reference IAP in patients undergoing laparoscopic cholecystectomy |
| Risin et al.  (2006) | Israel | 50 | 400 | To determine the efficacy of 14-Fr PVC round drain in the direct measurement of IAP | Prospective cohort study | Patients undergoing elective laparoscopic surgery | Direct measurement: a 14-Fr PVC round drain | Laparoscopic insufflator | r=0.996 | 1. Direct measurement of IAP using 14-Fr PVC round drain is a newly described technique that is simple, fast and credible 2. No complications were observed with this new technique |
| Risin et al.  (2006) | Israel | 40 | 440 | Explore if a 14-F polyvinyl chloride (PVC) round drain is a reliable tool for direct IAP measurement | Prospective cohort study | Patients undergoing abdominal surgery and treated postoperatively with intraperitoneal drains and intravesical catheters | Direct measurement: 14-F PVC round drain connected to an invasive blood pressure monitoring system | Intra-vesical method | r= 0.962 | 1. Direct measurement of the IAP via a 14-F round PVC drain is a feasible technique 2. Future investigation should be aimed at the confifirmation of the reliability of this technique for continuous monitoring of IAP in selected patients |
| Balogh  et al.  (2004) | Australia | 25 | 150 | To evaluate whether continuous IAP (CIAP) can be accurately measured via the irrigation port of a three-way catheter | Prospective cohort study | Surgical and trauma patients admitted to the ICU | Intra-vesical method:size 18-Fr standard three-way catheters (Lubri-Sil AllSilicone Foley catheter; C.R. Bard, Inc., Covington, GA) connected to a pressure transducer | Intra-vesical method:intermittent IAP measurement | Mean difference: 0.019±0.05 mmHg | 1. Continuous IAP measurement has an excellent   agreement with the IAP over wide pressure ranges   1. Continuous IAP measurement can be routine clinical used in general/vascular surgical patients after major abdominal surgery, in severe pancreatitis, in 15% total body surface area burns, and in trauma patients with severe traumatic shock and resuscitation or undergoing damage control laparotomy |
| Lee et al.  (2002) | USA | 30 |  | Describe a simple U-tube technique and investigate its accuracy for measuring IAP | Prospective cohort study | Patients undergoing laparoscopy | Intra-vesical method: using U-tube technique | Direct measurement: measured directly in the abdominal cavity | R^2^= 0.79 | The accuracy of the U-tube manometry technique for measuring  IAP is comparable to previously described techniques. The U-tube technique is simple, does not require additional equipment, and can be performed by any member of the medical team |
