# Supplementary material for: The development, feasibility and credibility of intra-abdominal pressure measurement techniques: A scoping review
Source: PLoS One. 2024 Mar 21;19(3):e0297982. doi: 10.1371/journal.pone.0297982 (PMC10956852; doi:10.1371/journal.pone.0297982)
Supplement: S2 Table — (DOCX) [file pone.0297982.s004.docx]

**S2 Table. Overview of the disadvantages (*****) of the different techniques for IAP measurement**

| **Categories** | **Sub-categories** | **No repeated measurements** | **No continuous trend** | **Not accurate** | **Not reproducible** | **Not well validated** | **Urinary infection** | **High cost of device** | **Invasiveness** | **Others** |
| --- | --- | --- | --- | --- | --- | --- | --- | --- | --- | --- |
| Direct  techniques | / |  |  |  |  |  |  | * | * | Validation should be performed in postoperative patients |
| Indirect techniques | Transvesical IAP measurement |  |  |  |  |  | * |  | * | Susceptible to factors such as body position, perfusion volume and bladder compliance |
|  | Transgastric  IAP measurement |  |  |  |  | * |  |  | * | It may affect nasal feeding and generate complications with traditional nasogastric tubes |
|  | Rectal IAP measurement | * |  | * | * | * |  |  | * | The failure rate of obtaining reproducible IAP values is very high |
|  | Intravaginal IAP measurement |  |  | * |  | * |  | * | * | The accuracy of this technique in measuring absolute IAP cannot be determined |
|  | Femoral venous IAP measurement |  |  | * | * | * |  | * | * | It is not reliable both in adult and pediatric ICU patients |
| Less invasive techniques | Abdominal wall tension IAP measurement |  | * |  | * | * |  |  |  | It is not standardized enough to obtain more reliable and repeatable evaluations |
|  | Microwave reflectometry IAP measurement |  |  | * | * | * |  | * |  | Measurement of absolute values of IAP using microwave refectometry remains a challenge |
|  | Ultrasonographic IAP measurement |  | * |  |  | * |  | * |  | The device has limited measurement resolution and can be used only as a relative measure or static guide for patient IAP |

**S2 Table. Overview of the advantages (-) of the different techniques for IAP measurement**

| **Categories** | **Sub-categories** | **Feasibility** | **Fast** | **Simple** | **Safe** | **Sensitivity** | **Consistency** | **Recommendations** |
| --- | --- | --- | --- | --- | --- | --- | --- | --- |
| Direct  techniques | / | * | * |  | * | * | * | It is not recommended as a routine method for IAP measurement and should be prioritized for critically ill patients where standard techniques are contraindicated or could be inaccurate |
| Indirect techniques | Transvesical IAP measurement | * | * | * | * | * | * | In most cases, intermittent measurement may be sufficient, continuous measurement is not suited for screening IAH, but is best for long-term continuous fully automated monitoring IAP, especially when patients with ACS need emergency abdominal decompression |
|  | Transgastric  IAP measurement | * | * | * | * | * | * | It is recommended to use when patients have a nasogastric tube but without Foley catheters in place or accurate bladder pressure is not possible |
|  | Rectal IAP measurement |  | * |  |  |  |  | It cannot be recommended as surrogate IAP measures |
|  | Femoral venous IAP measurement | * | * |  |  |  |  | It cannot be recommended as surrogate IAP measures |
|  | Intravaginal IAP measurement | * | * |  | * | * |  | It is not suitable for routine measurement of IAP and is mainly applicable to exploring the role of elevated IAP in the progression, recurrence, and incidence of pelvic floor disorder during exercise and daily activitiy |
| Less invasive techniques | Abdominal wall tension IAP measurement | * | * | * | * |  |  | A commercially available AWT measurement device may become a powerful tool for IAP standardized measurement and future studies should also clarify the effects of common factors, such as gender, BMI and muscle relaxants and mechanical ventilation on AWT in the ICU |
|  | Microwave reflectometry IAP measurement | * | * | * | * |  |  | Further research is necessary to optimize the sensitivity of this system |
|  | Ultrasonographic IAP measurement | * | * | * | * |  |  | Needing for further research in multi-center studies and more varied test subjects. Potential in dynamic testing and abdominal compliance measurement exist as future avenues for the technology |
