# Supplementary figures and images for: The development, feasibility and credibility of intra-abdominal pressure measurement techniques: A scoping review
Source: PLoS One. 2024 Mar 21;19(3):e0297982. doi: 10.1371/journal.pone.0297982 (PMC10956852; doi:10.1371/journal.pone.0297982)

**S1 Fig . PRISMA flow diagram for included studies selection**

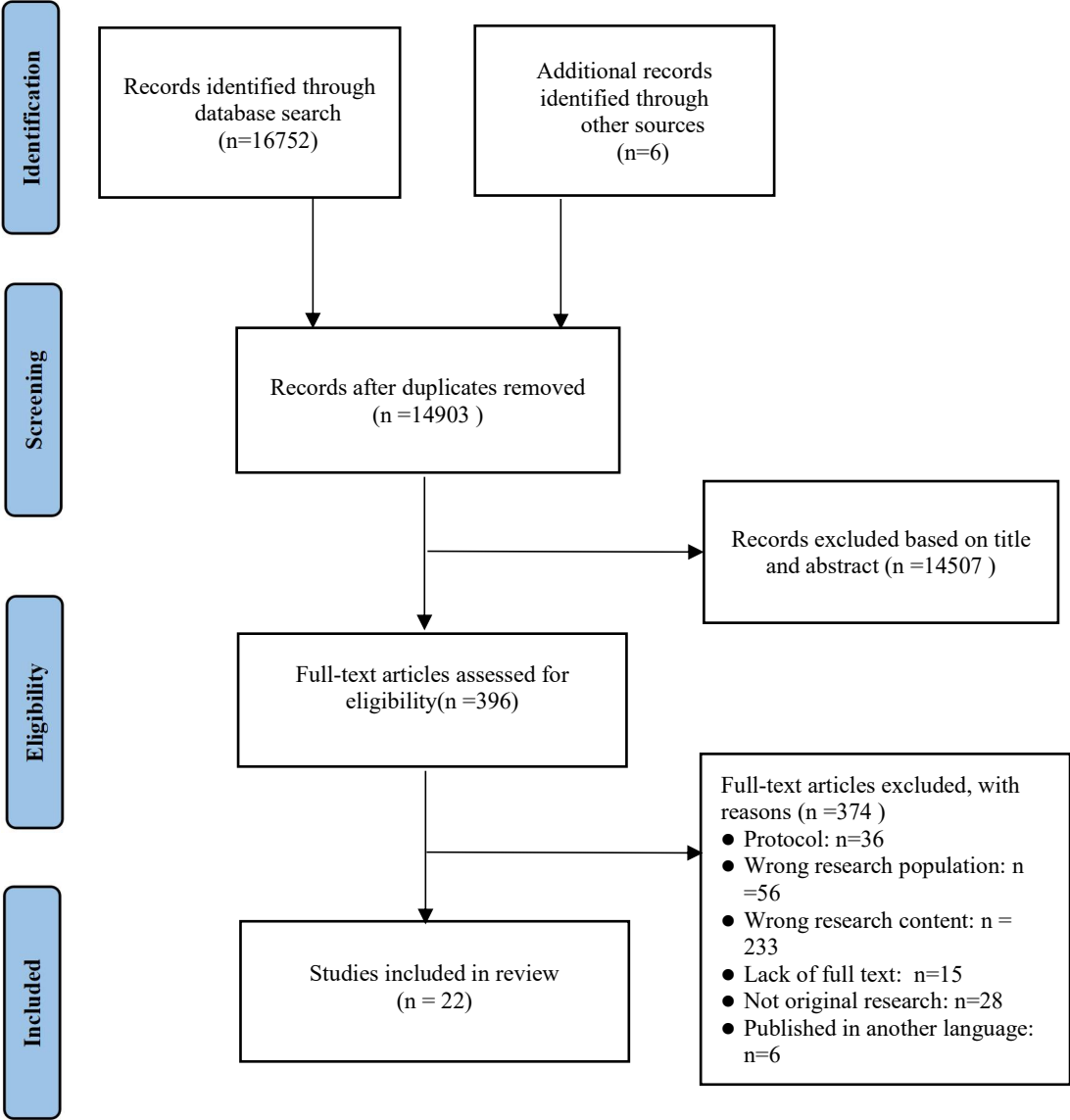

Supplement: S1 Fig — (PDF) [file pone.0297982.s005.pdf]
